# InteractomeSeq: a web server for the identification and profiling of domains and epitopes from phage display and next generation sequencing data

**DOI:** 10.1093/nar/gkaa363

**Published:** 2020-05-13

**Authors:** Simone Puccio, Giorgio Grillo, Arianna Consiglio, Maria Felicia Soluri, Daniele Sblattero, Diego Cotella, Claudio Santoro, Sabino Liuni, Gianluca De Bellis, Enrico Lugli, Clelia Peano, Flavio Licciulli

**Affiliations:** Laboratory of Translational Immunology, Humanitas Clinical and Research Center, IRCCS, Rozzano (Milan), 20089, Italy; Institute for Biomedical Technologies, National Research Council, Bari 70100, Italy; Institute for Biomedical Technologies, National Research Council, Bari 70100, Italy; Department of Health Sciences & Center for TranslationalResearch on Autoimmune and Allergic Disease (CAAD), Università del Piemonte Orientale, Novara 28100, Italy; Department of Life Sciences, University of Trieste, Trieste 34100, Italy; Department of Health Sciences & Center for TranslationalResearch on Autoimmune and Allergic Disease (CAAD), Università del Piemonte Orientale, Novara 28100, Italy; Department of Health Sciences & Center for TranslationalResearch on Autoimmune and Allergic Disease (CAAD), Università del Piemonte Orientale, Novara 28100, Italy; Institute for Biomedical Technologies, National Research Council, Bari 70100, Italy; Institute for Biomedical Technologies, National Research Council, Segrate (Milan) 20090, Italy; Laboratory of Translational Immunology, Humanitas Clinical and Research Center, IRCCS, Rozzano (Milan), 20089, Italy; Humanitas Flow Cytometry Core, Humanitas Clinical and Research Center, IRCCS, Rozzano (Milan) 20089, Italy; Institute of Genetic and Biomedical Research, UoS Milan, National Research Council, Rozzano (Milan) 20089, Italy; Genomic Unit, Humanitas Clinical and Research Center, IRCCS,Rozzano (Milan) 20089, Italy; Institute for Biomedical Technologies, National Research Council, Bari 70100, Italy

## Abstract

High-Throughput Sequencing technologies are transforming many research fields, including the analysis of phage display libraries. The phage display technology coupled with deep sequencing was introduced more than a decade ago and holds the potential to circumvent the traditional laborious picking and testing of individual phage rescued clones. However, from a bioinformatics point of view, the analysis of this kind of data was always performed by adapting tools designed for other purposes, thus not considering the noise background typical of the ‘interactome sequencing’ approach and the heterogeneity of the data. InteractomeSeq is a web server allowing data analysis of protein domains (‘domainome’) or epitopes (‘epitome’) from either Eukaryotic or Prokaryotic genomic phage libraries generated and selected by following an Interactome sequencing approach. InteractomeSeq allows users to upload raw sequencing data and to obtain an accurate characterization of domainome/epitome profiles after setting the parameters required to tune the analysis. The release of this tool is relevant for the scientific and clinical community, because InteractomeSeq will fill an existing gap in the field of large-scale biomarkers profiling, reverse vaccinology, and structural/functional studies, thus contributing essential information for gene annotation or antigen identification. InteractomeSeq is freely available at https://InteractomeSeq.ba.itb.cnr.it/

## INTRODUCTION

Protein biomarkers are fundamental in biomedicine, as they are pivotal tools for the diagnosis, prevention and treatment of diseases. Several techniques for the high-throughput screening and identification of protein interactions have been applied in biomarker discovery (1). In this scenario, phage display technology was introduced to identify short peptides with specific binding activity and subsequently evolved with many versatile applications, especially the display of antibodies (2). While it has been successfully exploited to select antibodies or peptides, the display of full-length proteins or protein domains expressed from libraries of cDNA (cDNA phage display) has been rarely used due to two main technical hurdles (3–5). First, the cloned cDNAs must be in the same reading frame as the phage coat protein and, when fused to the N-terminus, must not contain in-frame stop codons that would prematurely terminate the display of the fusion protein. Second, the display of full-length proteins is complicated because of the high degree of heterogeneity and complexity among the polypeptide sequences that must form functional fusions with the phage coat protein. These two problems have been addressed by the concept of ‘filtering’ DNA for open reading frames (ORF phage display), and several different approaches have been taken (6–12). The two major features common to these approaches is that DNA (either cDNA or the intron-less prokaryotic genomic DNA) is first randomly fragmented and DNA fragments with a homogeneous size (typically 300–1000 bp) are pooled and cloned into a ‘filtering’ vector. This vector contains a selectable marker (e.g. β-lactamase) downstream the cloning site, that allows the selection of the ORFs cloned in the correct frame. After applying the appropriate antibiotic selection pressure, the resulting library is composed, rather than full-length proteins, by properly folded protein domains, i.e. the so-called domainome. Such domain libraries are then fully functional and could undergo phage display selection cycle for the identification of specific interacting domains. With this approach, both eukaryotic and prokaryotic domain libraries obtained from genomes and transcriptomes can be analyzed. A filtered human domainome library has been positively used to profile the protein interactome of human tissue transglutaminase (8), to identify novel antigens in coeliac disease (12) and tumour-associated antigens in ovarian cancer (13) and to identify novel RNA-binding proteins relevant to the biology of AU-rich element (ARE) (14) or SINEUP long non-coding RNAs (15). Similarly genomic libraries from *Clostridium thermocellum* (16) and *Burkholderia pseudomallei* (17), have been constructed and successfully used for domain-based functional annotation purposes.

However, the output generated by ORF-filtering libraries sequencing cannot be analysed easily and adequately with the existing bioinformatics resources that have been designed and implemented for other purposes. In our previous papers (14,15,17,18), the analysis for the identification of specific domains/antigens was performed with NGS-TreX (19). NGS-TreX is a freely available web-tool designed for the analysis of of differential RNA-Seq data and it was not specifically developed for the analysis of Interactome-Sequencing data. In a recent work, Yang *et al.* (20)) adapted the CLC Genomics Workbench software to the analysis of this kind of data. Moreover, some pipelines under development are available in GitHub, such as for example nf-core/epitope prediction (21). However, up to date, there are not specific pipelines able to efficiently and reliably reveal enriched domains from datasets generated by Interactome-sequencing technology, especially there are no tools available as web servers.

Here, we propose a user-friendly web server implementing a workflow able to manage Interactome-sequencing data, with customizable parameters to perform specific testing for the identification of enriched domains/epitopes. At the same time, the web server implements an advanced visualization tool useful to highlight and share the identified domains. InteractomeSeq is freely available at https://InteractomeSeq.ba.itb.cnr.it/

## MATERIALS AND METHODS

**Figure 1.**
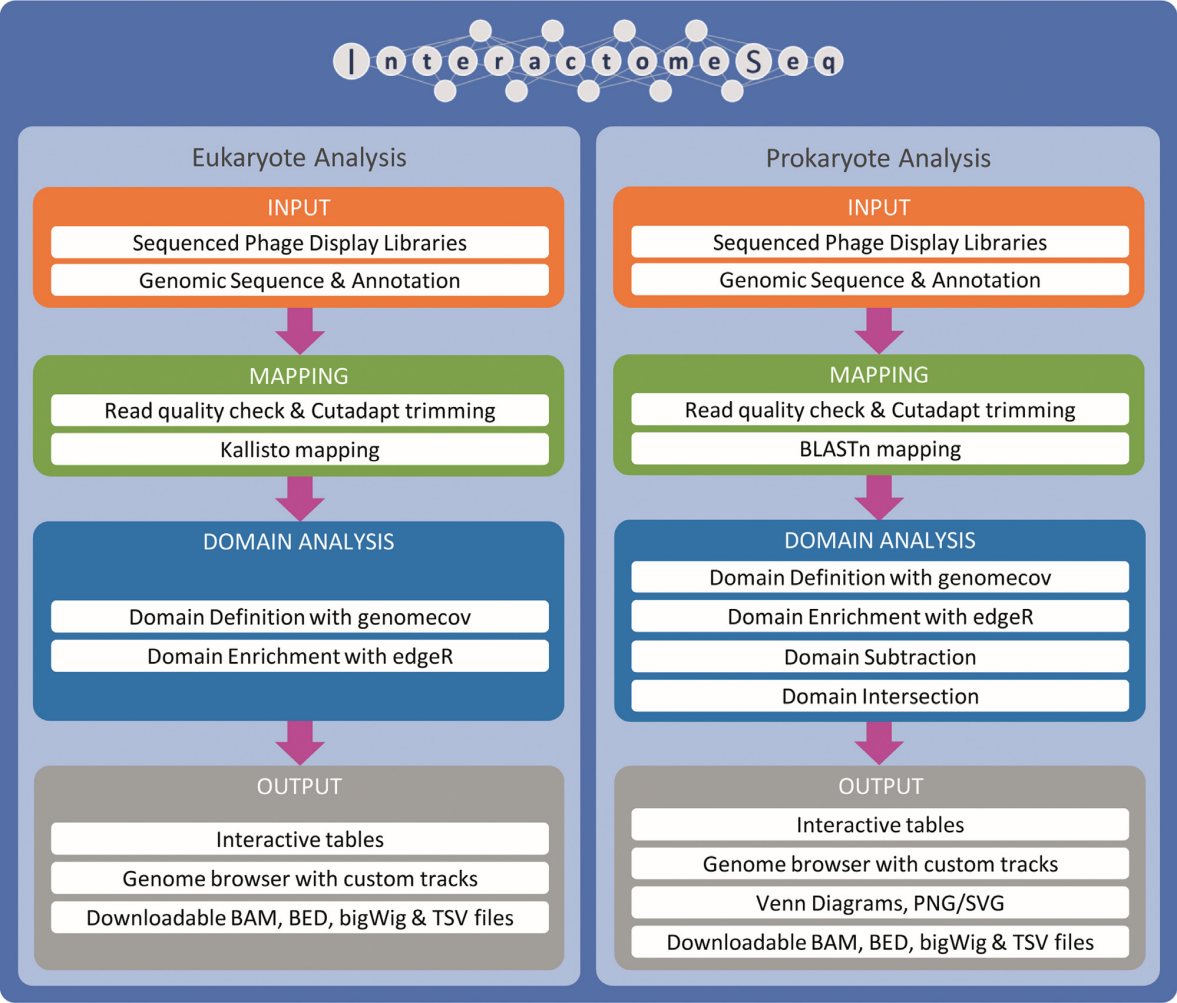
Overview of InteractomeSeq web server workflow. The tool implements two different workflows for Eukaryote and Prokaryote data. Prokaryote workflow includes two more steps for the Domain Analysis than the Eukaryote workflow. Moreover, the Domain Intersection step also produces a Venn diagram, among the other outputs.

InteractomeSeq, through a user-friendly web interface, implements a new pipeline allowing users to obtain an accurate characterization of domainome/epitome profiles. The architecture of InteractomeSeq consists of a Graphical User Interface on a series of Python scripts implementing two analysis pipelines, for Eukaryotic and Prokaryotic domainome analysis, as backend (see Figure 1).

### Description

InteractomeSeq web server is used to analyse data deriving both from phage libraries created from a whole genome/transcriptome and from phage libraries selected against different baits. In InteractomeSeq a complete domainome analysis is identified as a Project. Depending on the type of organism used to generate the phage libraries, in the ‘Create a Project’ page, users have to select Eukaryotic or Prokaryotic analysis type and define a Project Name. A Project ID, consisting of a unique 28-characters ID is created and associated with each Project, it can be used to access, resume and complete the analysis and visualize or download the results later. InteractomeSeq stores, on the server, the uploaded files and analysis data results in a user's private workspace accessible only via a RESTful web service, ensuring the user's privacy by a unique random key (Project ID). The main components of a Project are: Uploading, Mapping, Domain Analysis and Results. The execution of a complete analysis is split into each single execution step (Mapping, Domain Definition, Enrichment, Subtraction and Intersection) in an asynchronous mode, so the user can run, resume and re-run each analysis step in order to achieve the desired results. Through specific buttons, the user can monitor, eventually stop, re-run and delete the individual execution steps. The web tool displays the execution date, the input parameters, the message log and the results of each execution by clicking on the appropriate button. Finally, a colour-coding icon shows the running status of each execution. Detailed tutorials and guides on how to run the analysis steps in InteractomeSeq, are available in the Supplementary Data (Tutorials file) and in the Help page of the web tool, for Eukaryote and Prokaryote analysis type. The use of InteractomeSeq does not require registration, if users enter their email address (optional), they will receive a web link including the Project ID in order to be able to access their projects later. At the same time, the registration to the webtool gives to the users the possibility to manage their own list of analysis projects in a user's private session.

### Input files

The ‘Uploading’ page of the web tool allows users to upload the input files. The input files necessary to start a domainome analysis at whole genome/transcriptome level are the reads generated by the sequencing of genomic/transcriptomic phage libraries generated by following an interactome-sequencing approach (such as for example libraries produced following the protocol described by Soluri *et al.* (22)) and the reference genome/transcriptome of the target organism (i.e. bacteria, human, mouse). For the Prokaryote analysis type, a database of pre-loaded genome sequences and annotations is available, it includes all the bacteria complete genomes available in NCBI (ftp://ftp.ncbi.nlm.nih.gov/genomes/). This Database contains a total of 15 593 bacterial strains’ genomes (last updated November 2019). If the bacterial genome of interest is not present in the local database, the end user can upload a custom genome sequence and annotation in the ‘Custom Annotation’ section of the Uploading page. Sequence files in FASTA format and annotations in standard BED, GFF formats (see https://genome.ucsc.edu/FAQ/FAQformat for format specifications) or delimiter separated text files can be uploaded. Whereas for the Eukaryote analysis type the *Homo Sapiens* GRCh38 and *Mus Musculus* GRCm38 reference genomes/transcriptomes (https://www.ensembl.org/info/data/ftp/) are available within the InteractomeSeq internal database. The web tool checks the syntax correctness of the users’ provided annotation files and shows a preview of the content of all the annotation files that have been uploaded.

The raw sequencing reads can be uploaded in the DataSets section of the Uploading page. FASTA and FASTQ formats (preferably in gzip compress format) are allowed, long and short sequence data generated with single or paired-end library preparation kits are supported. In Eukaryotic analysis type, pre-aligned files in BAM format (https://genome.ucsc.edu/FAQ/FAQformat#format5.1) can also be uploaded. At least two samples or selections are required to perform the domain enrichment analysis. The maximum size of each uploaded file (Dataset) is set to 5GB (compressed file). Raw data files bigger than 5GB can be analyzed by the stand-alone version of the pipeline scripts available in GitHub or by the Docker version (see Data Availability).

### Mapping

The Mapping step creates the genome aligned BAM files from the sequencing reads of the phage libraries that have been uploaded. The web tool first validates the syntax of the input files, then raw reads are filtered by quality checking and are cleaned by anchor adapters using Cutadapt tools (23), while reads with no identifiable adapters are discarded. Trimmed reads are aligned to reference transcriptome/genome using BLASTn (24) or Kallisto (25), depending on prokaryotic or eukaryotic analysis type. Kallisto was chosen for the eukaryotic data mapping because it is one of the fastest aligners with better performance in terms of accuracy and memory requirement (26). However, Kallisto requires, as input, the Gene transfer format (GTF) file that is not available for all the prokaryotic genomes. Allowed mismatches (default 3) and minimum clone length (default 100) parameters are available in the mapping execution input form in order to tune the mapping procedure. Alignment files created by the mapping step are converted in bigWig format (https://genome.ucsc.edu/FAQ/FAQformat#format6.1) for visualization purposes.

### Domain analysis

The core of an analysis Project in InteractomeSeq are the steps located in the ‘Domain Analysis’ page. More than one selected phage library can be analysed at the same time, but the genomic/transcriptomic phage library dataset input is mandatory. The latter can be also analysed alone, if the focus of research is the identification of all potential soluble domains on a genomic/transcriptomic scale. In the ‘Domain Definition’ step, all the aligned BAM files are scanned for the putative domain detection using bedtools genomecov (27), then the predicted domains are assigned to their respective CDSs (more than one domain can be associated to one CDS) using bedtools intersect. In the ‘Domain Enrichment’ step, users can determine which putative domains are statistically enriched in the selected phage library sample compared to their representation in the genomic phage library (or not selected sample) which is used as reference/background. The differential enrichment is calculated using the R-package edgeR (28). In the Prokaryote analysis type, when more than two datasets from selected phage libraries are uploaded, differentially enriched domains in the different selections can be obtained through the subtracting (‘Domain Subtraction’) or intersecting (‘Domain Intersection’) steps, thus allowing the identification of those domains/antigens which are specific for different selections. In Eukaryote analysis type, common and unique putative domains are listed in the results of the ‘Domain Enrichment’ page. These lists of specific antigens are given in output as ranking lists associated with statistical values (adjusted p-value) thus allowing a guided selection of the best targets for validation (i.e. top list antigens).

### Output

**Figure 2.**
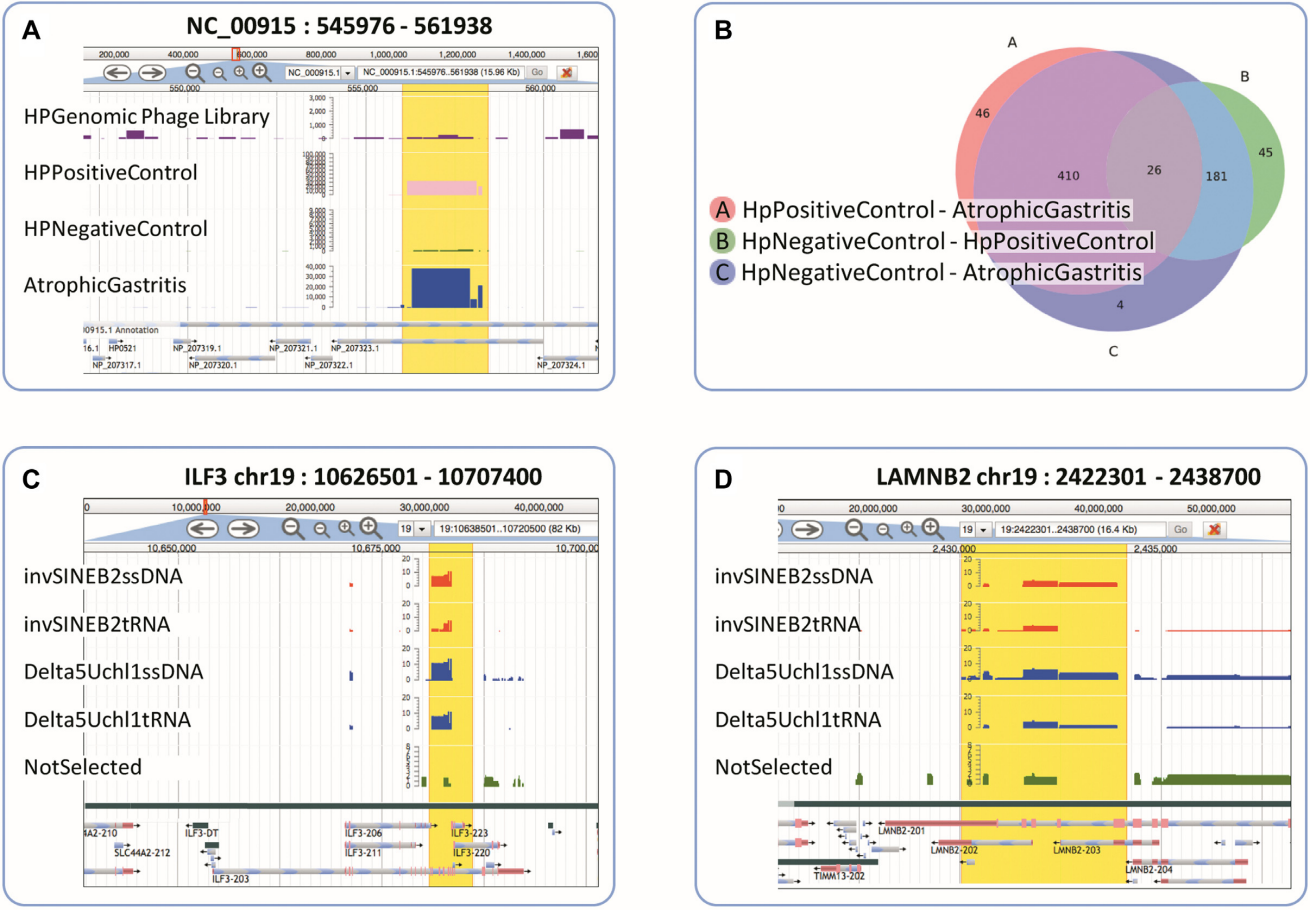
Example of outputs: (**A**) JBrowse visualization of the enriched domains of an *H. pylori* gene in the three different selections; (**B**) Venn Diagram showing the intersection among the *H. pylori* domains enriched in the different selections; (**C**) JBrowse visualization of the ILF3 domains enriched in the RNA Binding Protein (Eukaryote) dataset; (**D**) JBrowse visualization of the LMNB2 domains enriched in the RNA Binding dataset. Track colors in JBrowse can be personalized by changing the ‘pos_colour’ in ‘Edit config’. Panel A shows the tracks in arithmetic scale, by deselecting the ‘Log scale’ default.

InteractomeSeq provides results for each domain analysis step (Definition, Enrichment, Subtraction and Intersection). The final result is the list of putative domains, resulting enriched in the selected phage libraries respect to the genomic one. The lists are displayed in an interactive table viewer and as tracks in an embedded genome browser (JBrowse (29)) in order to display and visually compare the predicted domains and their relative abundance (see Figure 2). Domain chromosome location, gene name and transcript/gene location are shown in the table viewer, columns content can be alphabetically ordered and gene/transcript columns content can be filtered by text search. Further information, such as protein ID and region sequence are available in the metadata associated with each CDS and in the downloadable result files. Furthermore, for the Prokaryote analysis type, in the ‘Domain Intersection’ page an interactive Venn plot, downloadable as PNG and SVG file, shows the total of unique and common domains/epitopes that result from the intersection of two or three differentially enriched Selections. All the outputs are gathered in the ‘Results’ page of a Project and can be downloaded in compressed (zip) format. A Project is stored for 15 days and results are accessible or downloadable using a web link, containing the unique Project ID, which is reported in the ‘Information’ page. This page also reports the metadata about a project (Name, ID, type and dates) and the running status of each executed analysis step, in order to monitor the progress of the complete analysis.

### Implementation

The InteractomeSeq web server is based on a lightweight and flexible PHP Content Management System, named Typesetter CMS (https://www.typesettercms.com/), on the server side and on a specialized RESTful Web service, which manages the asynchronous communication with web interface. The web front-end, on the client side, is compliant with CSS3 and HTML5 standards adopting a Bootstrap Framework (http://getbootstrap.com) and is built upon Google AngularJS (http://angularjs.org/). A SQLite3 database is used to manage project metadata and user logging. Analysis pipelines are implemented in Python scripts, using various packages: (i) scientific libraries such as Numpy and pandas; (ii) bioinformatic packages such as BioPyhton, pybedtools and pysam; (iii) graphic library as Matplotlib. The scripts are available at: https://github.com/sinnamone/InteractomeSeq. InteractomeSeq is actually deployed on a server with 16-core CPUs (2.40 GHz), 64GB RAM and 20TB of storage.

## RESULTS

Three different use cases of InteractomeSeq are available as pre-computed analysis in the Examples box in the Home page and in the ‘Tutorials & Examples’ section of the Help page. The next paragraphs describe the aims and results of ‘Hp 26695 – Prokaryote’ and ‘RnaBindProt – Eukaryote’ use cases.

### Prokaryote analysis use case

InteractomeSeq has been used to define the whole domainome of *Helicobacter pylori* strain 26695 (HP 26695) and to outline new potential biomarkers of *H. pylori* infection and progression towards Atrophic Gastritis. Four different datasets have been analyzed: the genomic phage library obtained from the whole genome of *H. pylori* 26695 (label: 26695_S5) and three selected phage libraries. The last ones have been obtained after the selection of the HP 26695 genomic phage library against three pools of sera from patients: (a) sera from Healthy controls HP negative (label: HpNegativeControl); (b) sera from Healthy controls HP positive (label: HpPositiveControl); (c) sera from patients affected by atrophic gastritis (label: AthrophicGastritis) (see DataSets section in the ‘Uploading’ page,).

InteractomeSeq is able to outline all the *H. pylori* domains that are represented within the genomic phage library and, by analysing the selected phage libraries, to identify the *H. pylori* domains potentially actively interacting with patients’ antibodies (see ‘Domain Analysis’ page – Domain Definition List). Then, domains/epitopes enriched specifically in the different selections, specific of the healthy condition or of the atrophic gastritis outcome, or common to different conditions, are obtained (see ‘Domain Subtraction’ page – Domain Subtraction List) and summarized in the Venn Diagram (see ‘Domain Intersection’ page) (see Figure 2A and B). The InteractomeSeq pipeline allowed the identification of many specific HP domains/epitopes that can be validated in the future and possibly become new biomarkers of *H. pylori* infection and progression.

### Eukaryote analysis use case

By profiling the RNA interactome of a SINEUP long non-coding RNA, Fasolo *et al.* (15) recently reported a specific interaction between the RNA-binding protein ILF3 and the SINE B2 family of transposable elements in the mouse and human transcriptomes. In this paper, the datasets deriving from phage libraries sequencing were analyzed with NGS-Trex (19). We have analyzed the same datasets with InteractomeSeq and we have compared the results, focusing in particular on the mapping performance and on epitopes prediction to check if InteractomeSeq performs better than NGS-Trex. As shown in the Supplementary Table, the mapping accuracy of InteractomeSeq is on average 2–3% higher, because of the use of Kallisto mapper, which implements the novel concept of pseudo-alignment of reads for accurate quantification. Regarding the analysis results, it is important to underline that the number of genes with at least one putative domain detected is consistently higher in InteractomeSeq compared to those outlined by NGS-Trex and a wider number of putative domains/epitopes are predicted (see Supplementary Table). From a biological point of view, it is important to pinpoint that the results obtained with InteractomeSeq confirmed ‘IL enhancer-binding factor 3’ (ILF3) as a protein partner of ‘AS Uchl1 RNA’ (see Figure 2C), giving at the same time a clear overview of the interacting domains of the gene/protein. Moreover, using InteractomeSeq, we were able to predict a new potentially interesting RNA-binding protein interactor: the gene LMNB2, encoding for Lamin B2. Within this gene/protein two possible putative interacting domains were identified, as shown by the JBrowse panel in Figure 2D. Lamin B2 maintains chromosome integrity by ensuring proper spindle assembly and a decrease in Lamin B2 expression has been associated with chromosomal instability in colorectal cancer cell lines (30). A recent paper (31) shows that the nuclear Lamin B2 (Lmnb2) expression is essential for karyokinesis in mammalian cardiomyocytes and heart regeneration. Up to date, LaminB2 has never been considered a potential RNA binding protein.

## DISCUSSION

More than 20 computational methods for analyzing phage display next generation sequencing data, for reporting target-unrelated peptides (TUPs) and for predicting epitopes have been reviewed by He *et al.* (32). Some of the most relevant bioinformatics tools that have been used to analyze data deriving from Phage Display libraries sequencing include RELIC (33), PEPTIDE (34), DNAStar (35), SiteLight (36) and SLimFinder (37). These tools allow motif detection and epitope alignment but have been designed for the analysis of a limited number of sequences and they do not allow the comparison of different samples. On the other hand, PHASTpep (38), PepSimili (39), PuLSE (40) and NGS-Trex (19) have been used to analyze large datasets deriving from the high throughput sequencing of Phage Display libraries and allow the comparison between different samples and selections. PHASTpep is a MATLAB software that has been used for the discovery of cell-selective peptides. The software code is freely available but a graphical user interface is not available neither the pipeline was included in a web server. NGS-Trex has been widely used for the analysis of phage display library datasets (12,14,15,17). It was originally designed for RNA-Seq analysis and the result file shows a list of genes statistically enriched and sorted according to a parameter called ‘Focus’, but NGS-Trex is not able to predict the presence of multiple domains/epitopes present in each gene/protein and represented within the phage library analysed. At the same time, its output table lacks of the amino acid or nucleotide sequence of the domain, thus it is not possible to perform downstream analysis at the protein level. Vekris *et al.* (39) recently published a new computational galaxy pipeline, PepSimili, an integrated workflow tool, which performs mapping of massive peptide repertoires obtained by high throughput sequencing of phage display libraries on whole proteomes and delivers a streamlined systems-level biological interpretation. PepSimili pipeline has a user-friendly interface, but only one sample at a time could be analysed (i.e. one control sample against one test selection), thus the performance of this tool is limited when many different controls and/or selections should be analysed. Furthermore, it is not clear if both PHASTpep and PepSimili could analyse prokaryotic data. PuLSE (Phage Library Sequence Evaluation) (40) is a tool for assessing randomness and therefore diversity of phage display libraries. PuLSE is a useful pipeline that allows performing the evaluation required for QC of phage library randomness by NGS data to determine the positional and overall distribution of DNA bases and resultant amino acid propensities, calculating enrichment factors over the expected ideal. It is freely available from https://github.com/stevenshave/PuLSE as a free open source package. Another useful pipeline was developed for assessing phage display library diversity, and to investigate the bias in GE-libraries of linear, macrocyclic and chemically post-translationally modified (cPTM) tetrapeptides displayed on the M13KE platform, it was implemented as a MATLAB workflow by He *et al.* (41). However, the last two tools are not currently available as user-friendly web servers.

The InteractomeSeq web server overcomes all the main limitations showed by the previous bioinformatics tools for the analysis of high throughput phage display libraries:

1. It can analyze both Prokaryotic and Eukaryotic phage display libraries sequencing data (15593 bacterial strains’ genomes and the *H. Sapiens* GRCh38 and *M. Musculus* GRCm38 genomes are available).
2. It allows the identification of more than one putative domain/epitope within each gene/protein. The ‘blind’ detection of putative domains is very important to reduce the noise background typical of approaches based on phage display coupled with NGS. Moreover, the identification of domains/epitopes enables the precise definition of the interacting portions of target proteins and/or the specific epitopes recognized by antibodies.
3. InteractomeSeq results can be easily managed in an interactive tabular display and efficiently visualized through the JBrowse tool, which allows custom searches of the genes of interest and quickly navigate across the genome/transcriptome analyzed.
4. Many different datasets can be analyzed at the same time and in an asynchronous way, thus allowing the comparison among different selections performed by using the same phage library against different baits. Moreover, the intersection of enriched domains derived from more than one selection can be easily visualized through an interactive Venn diagram.
5. The computational resources required for the execution of InteractomeSeq are proportional to the size of the input files elaborated at each step (both raw data and reference sequence). In particular, the most memory-consuming step is the Mapping, because it loads the reference genome in the RAM and it is strictly related to the size of the reference genome. For example, in the Eukaryote analysis, the RAM occupied by the mapping step is about 3.7 GB (human genome), while the RAM used by the Prokaryote analysis for *H. pylori* genome is about 300 MB for each BLASTn thread. The RAM used by the Domain Analysis steps is proportional to the size of the input raw data files (Datasets) and is generally included in the upper bound reached by the Mapping step. The runtime of the pipeline depends on the size of the input files and on how many steps the user decides to run simultaneously, as well as on the hardware characteristics of the server. In particular, for the full analysis of the RnaBindProt (Eukaryote) analysis, InteractomeSeq takes about 40 minutes, while in the Hp 26695 (Prokaryote) example it takes about 30 minutes. In the Supplementary Data tutorials we show the size of each input file and a table summarizing the runtime of each step and the size of the output files.

In the previous Results paragraph, we have described two main applications of InteractomeSeq that are proposed in the webtool as examples: the first application is related to the definition of the whole domainome of *H. pylori* strain 26695 (HP 26695) and to the identification of new potential biomarkers of *H. pylori* infection and progression towards Atrophic Gastritis. The second application is related to the identification of new RNA functions and their functional annotation through the recognition of the proteins with which they form specific complexes. Interactome-sequencing technique has been used to profile RNA–protein interactions in a genome-wide manner in humans by analysing the datasets of Fasolo *et al.* By using InteractomeSeq we have demonstrated that new potential RNA-binding proteins and their specific domains interacting with new RNA classes can be outlined, as in the case of Lamin B2. Thus, we have demonstrated that InteractomeSeq can be successfully applied in different fields of research and that it can give very useful and reliable results. In particular, it is worth remarking that in the study of RNA-binding proteins, InteractomeSeq web tool is particularly powerful when many RNAs interact with the same protein, because it allows to identify different domains in the same RNA binding protein potentially interacting with different RNAs, thus enacting the guilt-by-association logic to infer their function. At the same time, with this approach, it is possible, for example, to identify groups of functionally related transcripts commonly associated with RNA-binding hub proteins.

In conclusion, the release of this tool will provide relevant support for the scientific and clinical community, because InteractomeSeq will fill an existing gap in the field of large-scale biomarkers profiling, reverse vaccinology, structural/functional studies, and discovery of new RNA-binding proteins and new transcript functions, thus contributing essential information for antigen identification or genome/transcriptome annotation.

## DATA AVAILABILITY

Pipeline source code, implemented in Python, is freely available for download at GitHub: https://github.com/sinnamone/InteractomeSeq. A Docker image is available in the Docker Hub public repository at https://hub.docker.com/r/flavioli/interactomeseq. The image contains the deployed version of the scripts available in GitHub and the Conda environment, in order to run InteractomeSeq pipelines on personal/private or big datasets.

The input datasets of the use cases described in the paper and analyzed in the Examples page are available in ENA with the following BioProject accession numbers: PRJEB37162 for Hp 26695 (Prokaryote); PRJEB37161 for RnaBindProt and RIDome (Eukaryote).

## Supplementary Material

gkaa363_Supplemental_Files
