# Supplementary material for: InteractomeSeq: a web server for the identification and profiling of domains and epitopes from phage display and next generation sequencing data
Source: Nucleic Acids Res. 2020 May 13;48(W1):W200–7. doi: 10.1093/nar/gkaa363 (PMC7319578; doi:10.1093/nar/gkaa363)

InteractomeSeq Examples

Tutorials

Prokaryote - Tutorial Hp 26695 2
Input Files: dataset Prokaryote_Hp26695.zip 3
Create a project 4
Uploading 5
Mapping 7
Domain Analysis 10
Results 18
Eukaryote - Tutorial RnaBindProt 19
Input Files: dataset Eukaryote_RnaBindProt.zip 20
Create a project 21
Uploading 22
Mapping 24
Domain Analysis 28
Results 32

Prokaryote - Tutorial Hp 26695

This introductory section provides an overview of **Prokaryote** pipeline drafting and design. The vertical gray rectangles correspond to the website sections.

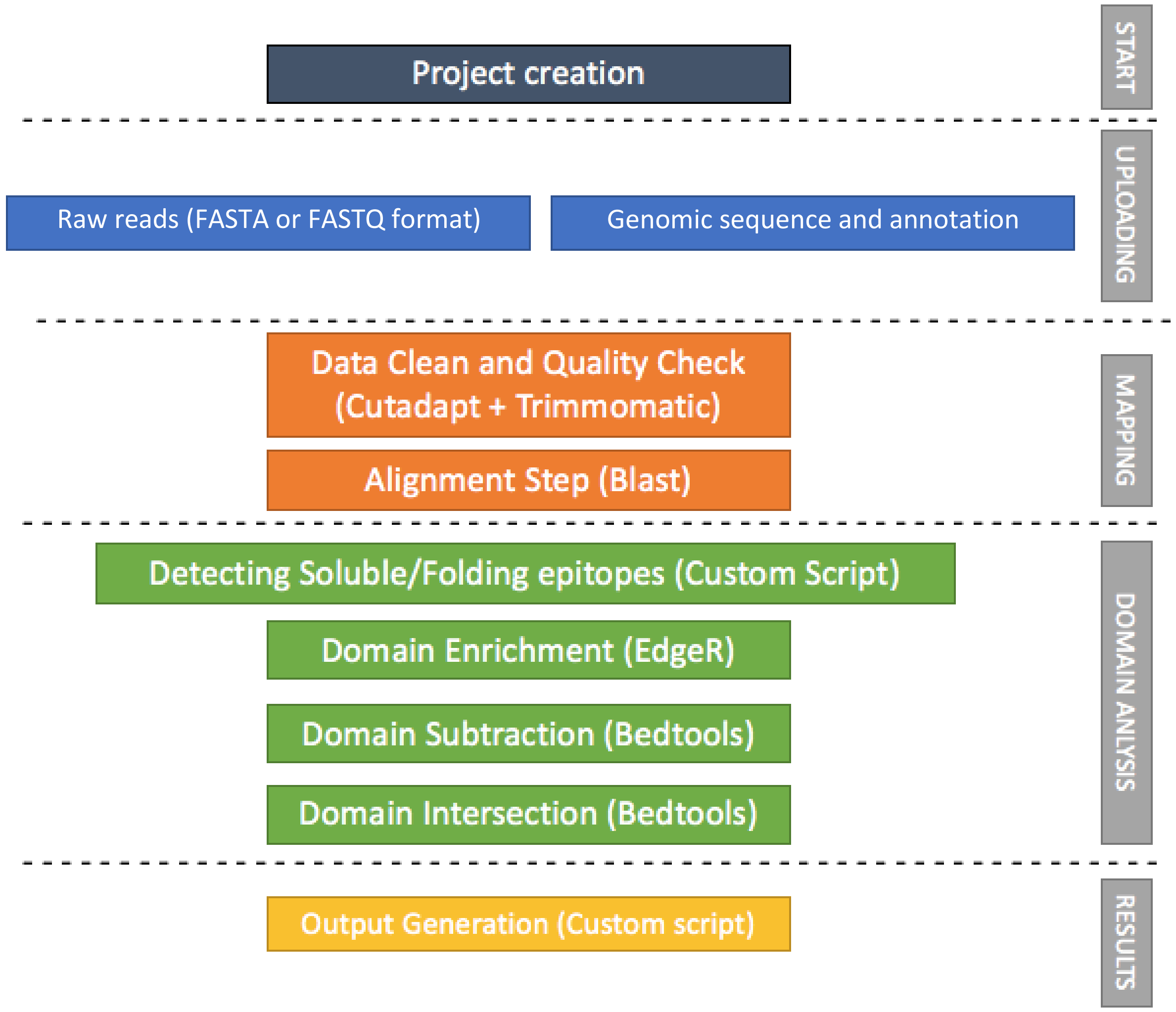

This document shows how to perform all the steps of an example analysis with the data provided for Prokariote pipeline. For further information about the forms and the interpretation of the results, refer to the User Guide.

Input Files: dataset Prokaryote_Hp26695.zip

[Download a zip folder containing the raw datasets from this link (1.1 GB).]

Mandatory inputs for **InteractomeSeq - Prokaryote Hp 26695** execution are:

- genome reference file in FASTA format (either a custom annotation file or one selected from the drop-down menu) (Organism).
  - GCF_000008525.1_ASM852v1_genomic.fna (1.6 MB)
- a genome annotation (either a custom annotation file or one selected from the drop-down menu) (Organism).
  - GCF_000008525.1_ASM852v1_genomic.gff (620 KB)
- Raw Data files, FASTA or FASTQ format for query reads are allowed in the input, therefore the web interface additionally allows the submission of compressed files (gz format) to reduce the time of data upload (DataSets).
  - HP_genomic_26695_R1.fastq.gz (97 MB)
  - HP_genomic_26695_R2.fastq.gz (133 MB)
  - Sel_AG_R1.fastq.gz (243 MB)
  - Sel_AG_R2.fastq.gz (335 MB)
  - Sel_HPneg_R1.fastq.gz (66 MB)
  - Sel_HPneg_R2.fastq.gz (91 MB)
  - Sel_HPpos_R1.fastq.gz (74 MB)
  - Sel_HPpos_R2.fastq.gz (104 MB)

InteractomeSeq requires the user to upload at least two datasets. The input datasets must be generated with the same sequencing platform.

Time and memory usage for this tutorial

Analysis Step	File	Excution time	Output size
Mapping	26695_S5	~ 9m	46 MB
Mapping	HpNegativeControl	~ 6m	8 MB
Mapping	HpPositiveControl	~ 12m	13 MB
Mapping	AtrophicGastritis	~ 28m	28 MB
Domain Definition	26695_S5	~ 2m	357 KB
Domain Definition	HpNegativeControl	~ 35s	84 KB
Domain Definition	HpPositiveControl	~ 50s	126 KB
Domain Definition	AtrophicGastritis	~ 1m	222 KB
Domain Enrichment	26695_S5 - HpNegativeControl	~ 20s	30 KB
Domain Enrichment	26695_S5 - HpPositiveControl	~ 25s	39 KB
Domain Enrichment	26695_S5 - AtrophicGastritis	~ 30s	82 KB
Domain Subtraction	HpPositiveControl - AtrophicGastritis	< 5s	52 KB
Domain Subtraction	HpNegativeControl - HpPositiveControl	< 5s	27 KB
Domain Subtraction	HpNegativeControl - AtrophicGastritis	< 5s	67 KB
Domain Intersection	HpPositiveControl - AtrophicGastritis	< 5s	97 KB
Domain Intersection	HpNegativeControl - HpPositiveControl - AtrophicGastritis	< 5s	122 KB

Create a project

Click the button **START** to create a new project. Give the project a name (mandatory), a description (optional) and an email address (to receive messages during the execution. Select **PROKARIOTE** as project type and **SAVE**.

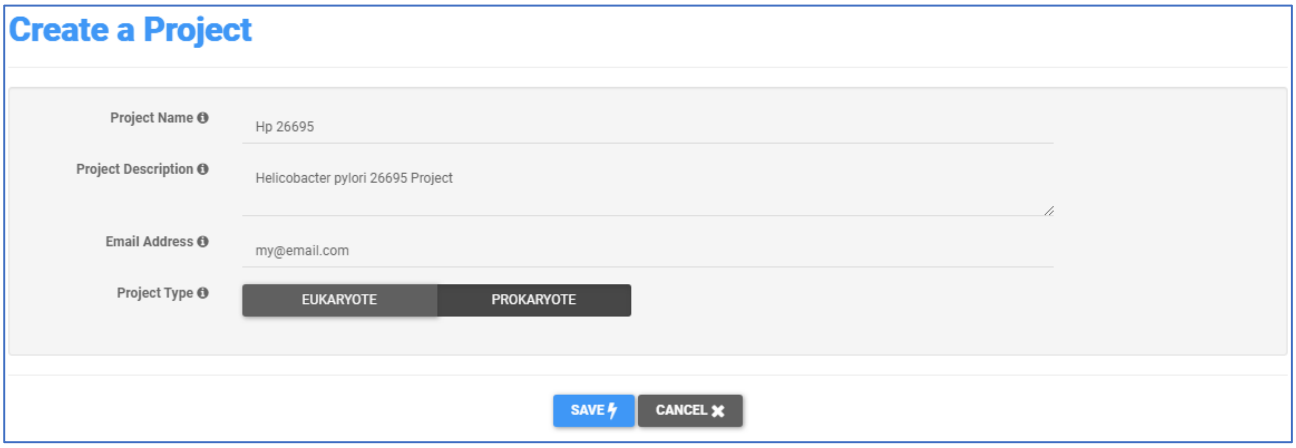

The project now has an ID that is a link, active for 15 days. Save this link if you didn't provide an email address (the link will be sent via email).

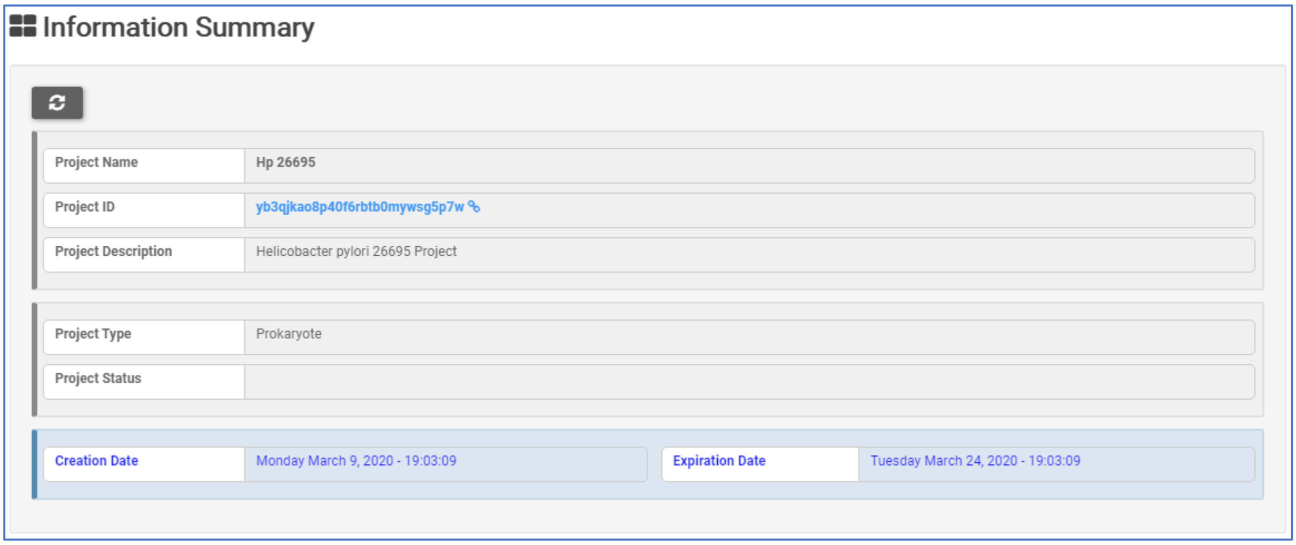

Uploading

Clik on **UPLOADING** to upload the FASTQ files.

Select the **Custom Annotation** form to upload the genome files. Upload the reference file (**GCF_000008525.1_ASM852v1_genomic.fna**), and press **VALIDATE**. After the validation, a preview of the genomic sequences will be shown in a table.

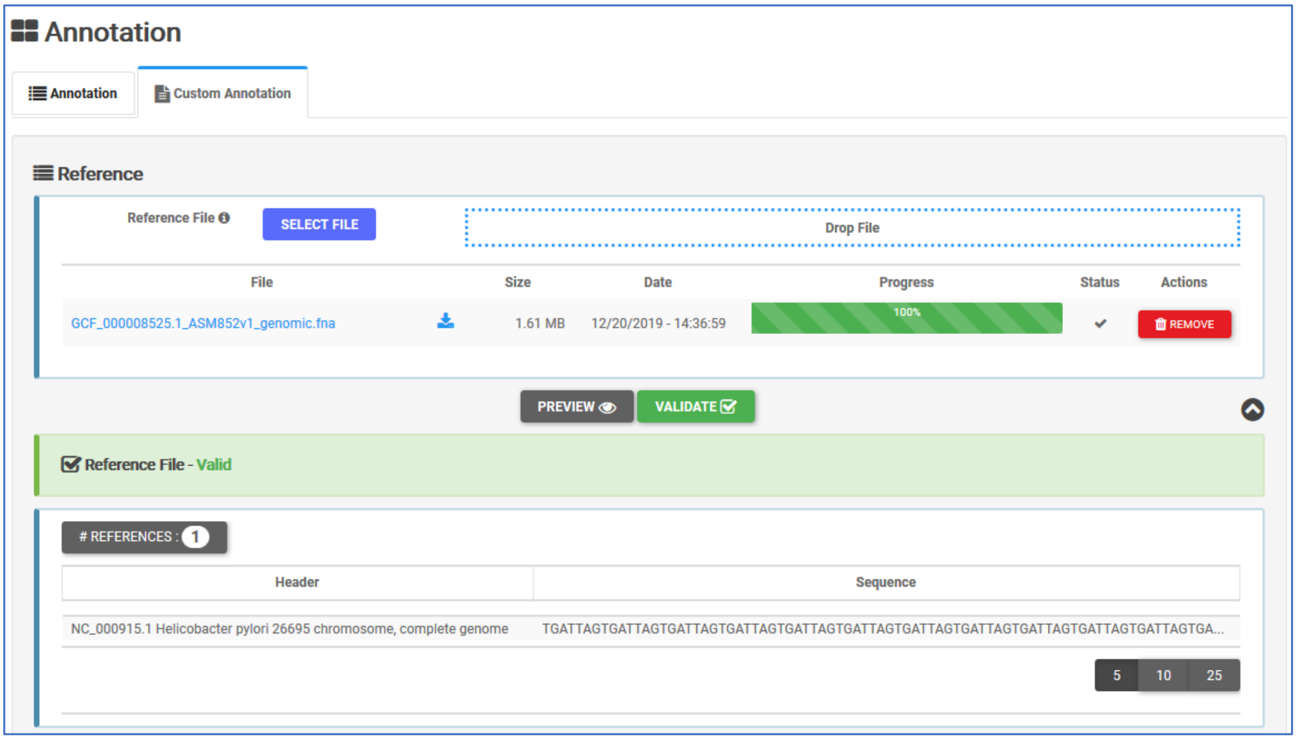

Upload the annotation (**GCF_000008525.1_ASM852v1_genomic.gff**), select the annotation format (**GFF**) and press **VALIDATE** (it is not necessary to compile the other fields, since GFF is a standard format).

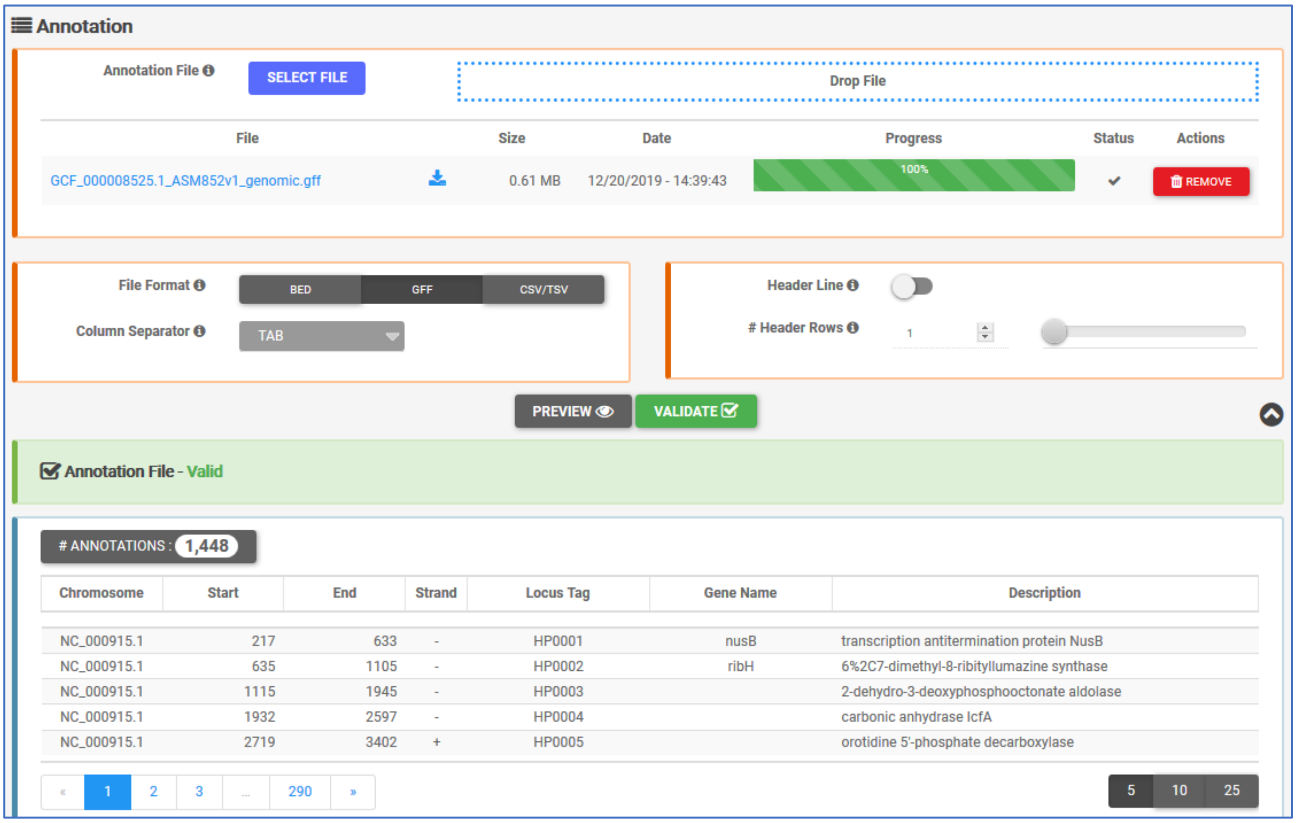

In the **DataSets** section, click on **SELECT FILE** or drag&drop files to upload FASTQ datasets:

- HP_genomic_26695_R1.fastq.gz
- HP_genomic_26695_R2.fastq.gz
- Sel_HPneg_R1.fastq.gz
- Sel_HPneg_R2.fastq.gz
- Sel_HPpos_R1.fastq.gz
- Sel_HPpos_R2.fastq.gz
- Sel_AG_R1.fastq.gz
- Sel_AG_R2.fastq.gz

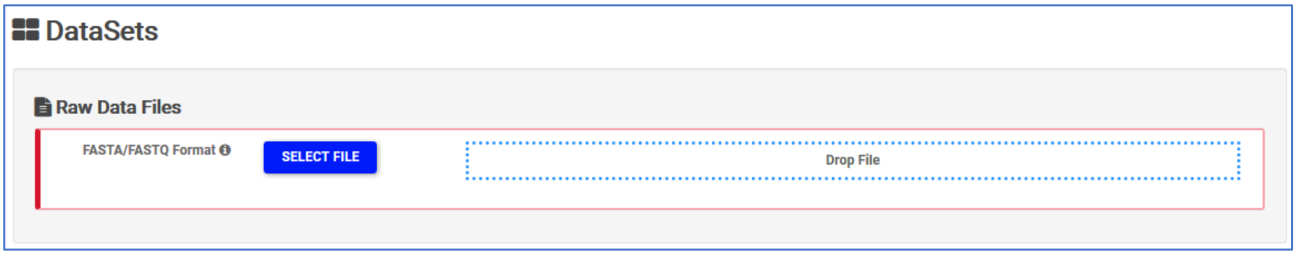

Dataset loading visualization:

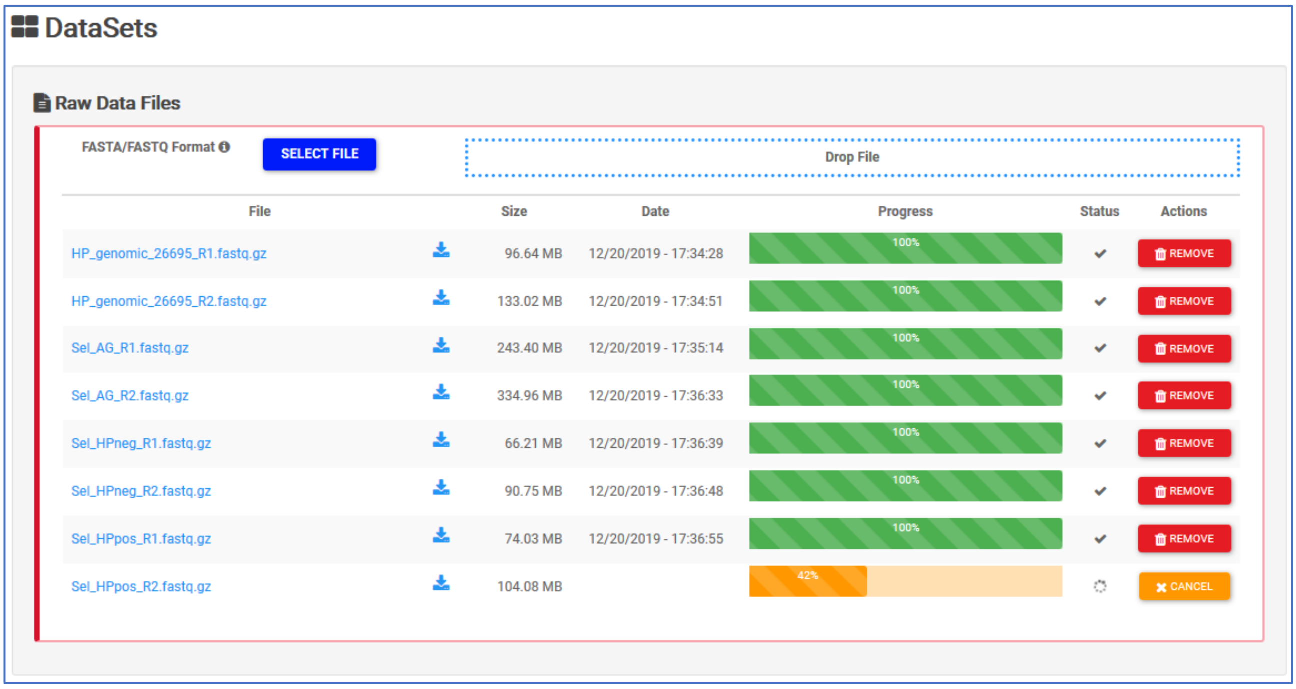

Mapping

In the **MAPPING** section, by clicking on the button **+ MAPPING,** 4 sub-sections will appear on the screen:

1. **Mapping Params.** Select paired-end reads (in this example: HP_genomic_26695_R1.fastq.gz and HP_genomic_26695_R2.fastq.gz)

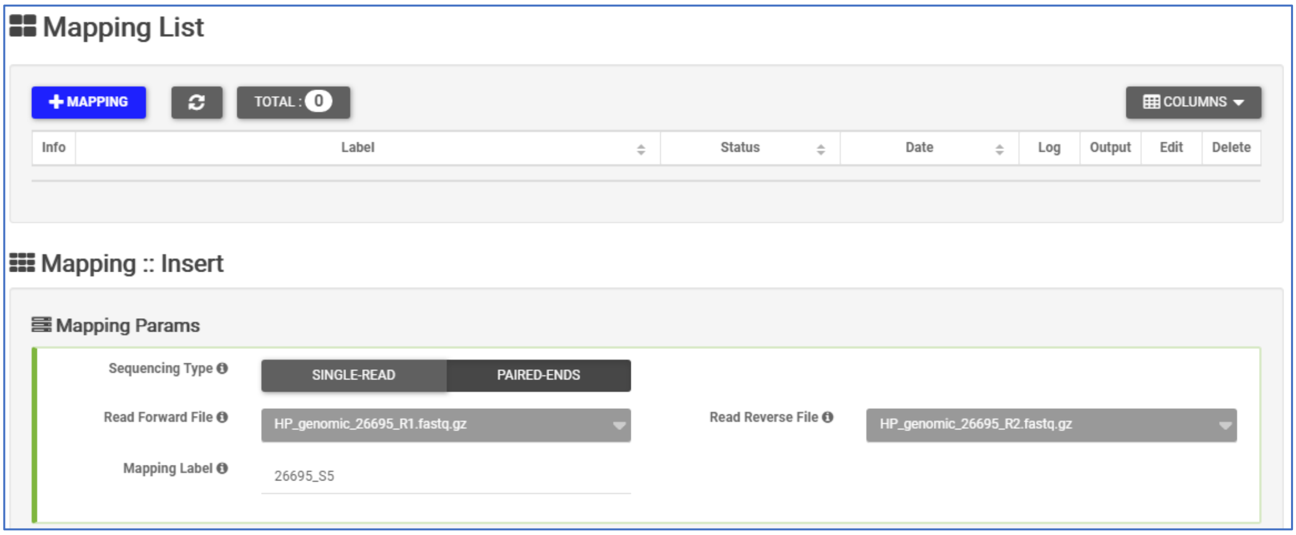

2. **Organism.** Preloaded FASTA file that will be used as reference to align the sequences.

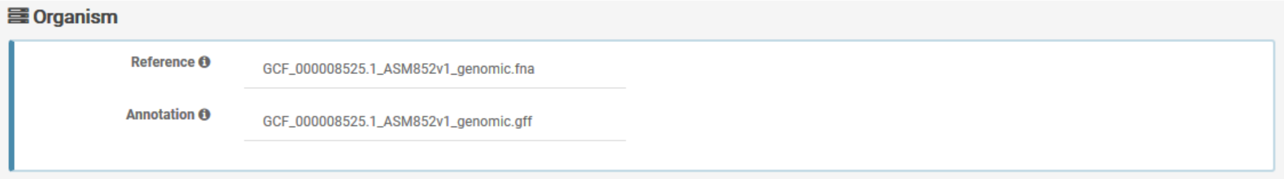

3. **Adapters.** Select **Custom Adapters** and insert:
   a. Forward Read 5' Adapter: GCAGCAAGCGGCGCGCATGCCACTAGTGGGAT
   b. Forward Read 3' Adapter: CCCAGAGCAA
   c. Reverse Read 5' Adapter: GGGATTGGTTTGCCGCTAGCGGAGAT
   d. Reverse Read 3' Adapter: CCCAGAGCAA

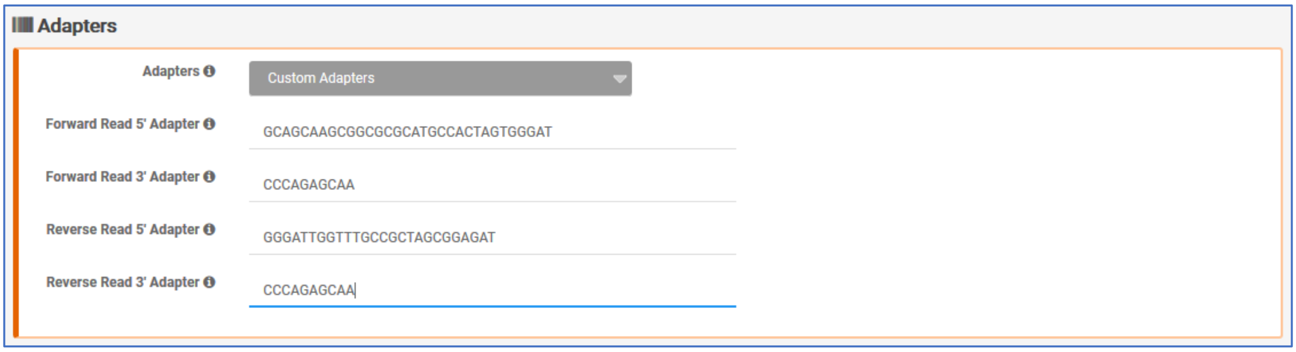

4. **Trimming Params.** Selection of minimum length of sequence and number of mismatch allows, reads below this threshold will be discarded. Use the default, and click **EXECUTE**

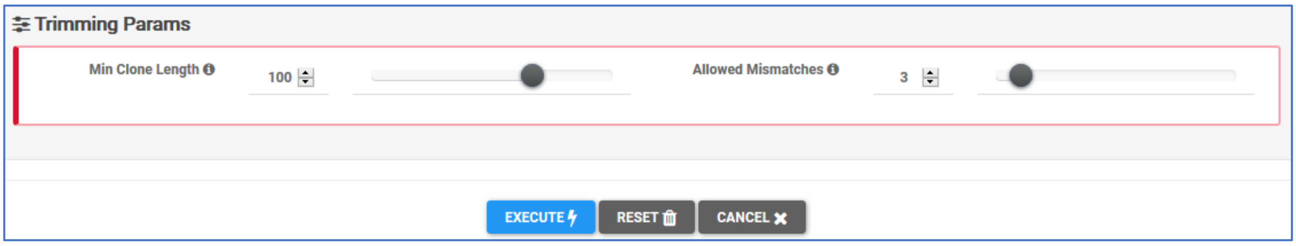

This mapping will appear in the list of running mappings.

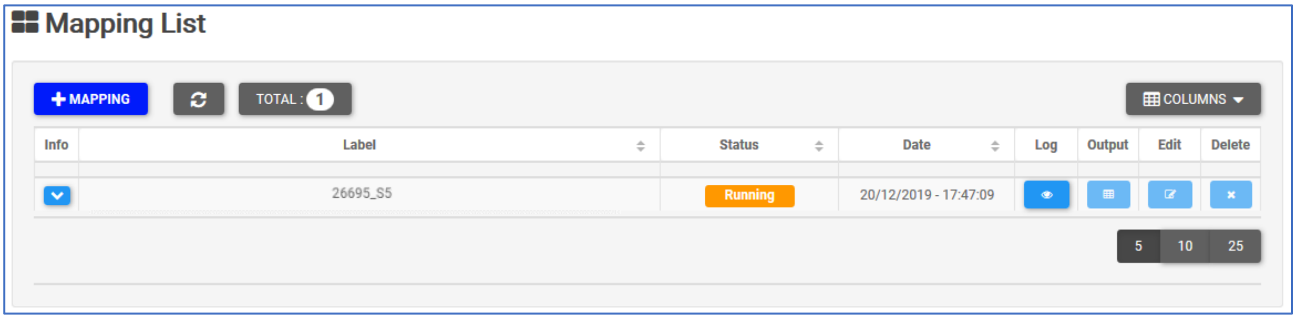

Here are the parameters for the other 3 mappings in this example:

1. Sel_HPneg_R1

    SequencingType Paired-Ends

    Forward Read File Sel_HPneg_R1.fastq.gz

    Reverse Read File Sel_HPneg_R2.fastq.gz

    Reference GCF_000008525.1_ASM852v1_genomic.fna

    Annotation GCF_000008525.1_ASM852v1_genomic.gff

    AdapterType Custom

    Forward Read 5' Adapter GCAGCAAGCGGCGCGCATGCCACTAGTGGGAT

    Forward Read 3' Adapter CCCAGAGCAA

    Reverse Read 5' Adapter GGGATTGGTTTGCCGCTAGCGGAGAT

    Reverse Read 3' Adapter CCCAGAGCAA

    Min Clone Length 100

    Allowed Mismatches 3

2. Sel_HPpos_R1

    SequencingType Paired-Ends

    Forward Read File Sel_HPpos_R1.fastq.gz

    Reverse Read File Sel_HPpos_R2.fastq.gz

    Reference GCF_000008525.1_ASM852v1_genomic.fna

    Annotation GCF_000008525.1_ASM852v1_genomic.gff

    AdapterType Custom

    Forward Read 5' Adapter GCAGCAAGCGGCGCGCATGCCACTAGTGGGAT

    Forward Read 3' Adapter CCCAGAGCAA

    Reverse Read 5' Adapter GGGATTGGTTTGCCGCTAGCGGAGAT

    Reverse Read 3' Adapter CCCAGAGCAA

    Min Clone Length 100

    Allowed Mismatches 3

3. Sel_AG_R1

    SequencingType Paired-Ends

Forward Read File Sel_AG_R1.fastq.gz

Reverse Read File Sel_AG_R2.fastq.gz

Reference GCF_000008525.1_ASM852v1_genomic.fna

Annotation GCF_000008525.1_ASM852v1_genomic.gff

AdapterType Custom

Forward Read 5' Adapter GCAGCAAGCGGCGCGCATGCCACTAGTGGGAT

Forward Read 3' Adapter CCCAGAGCAA

Reverse Read 5' Adapter GGGATTGGTTTGCCGCTAGCGGAGAT

Reverse Read 3' Adapter CCCAGAGCAA

Min Clone Length 100

Allowed Mismatches 3

This is the list of running mappings:

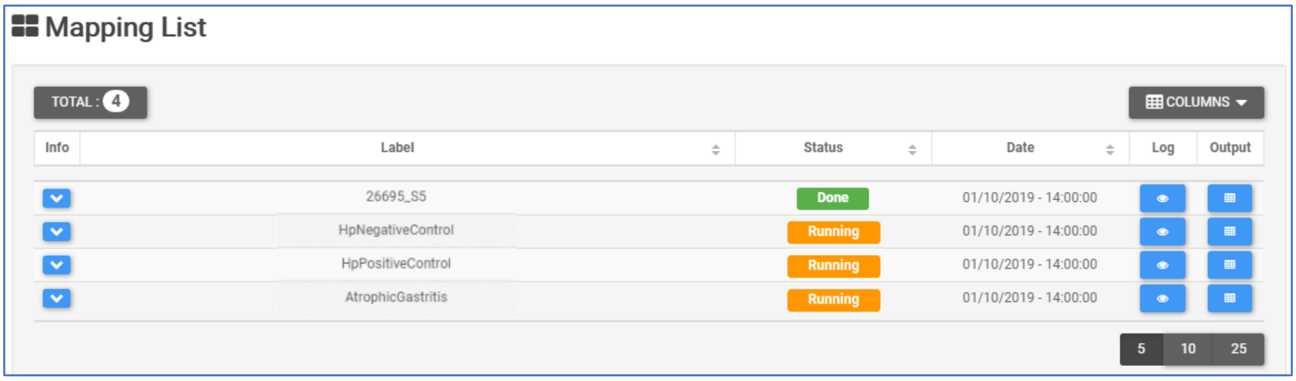
Mapping List

TOTAL : 4 | COLUMNS

Info	Label	Status	Date	Log	Output
	26695_S5	Done	01/10/2019 - 14:00:00		
	HpNegativeControl	Running	01/10/2019 - 14:00:00		
	HpPositiveControl	Running	01/10/2019 - 14:00:00		
	AtrophicGastritis	Running	01/10/2019 - 14:00:00		

5 10 25

Domain Analysis

Domain analysis is composed by four sheets:

1. **Domain Definition**
2. **Domain Enrichment**
3. **Domain Subtraction**
4. **Domain Intersection**

Domain Definition takes as input the mapping file previously generated. Define the domains for each of the 4 datasets uploaded and mapped by choosing them in the **Mapping** menu, then press **Execute**.

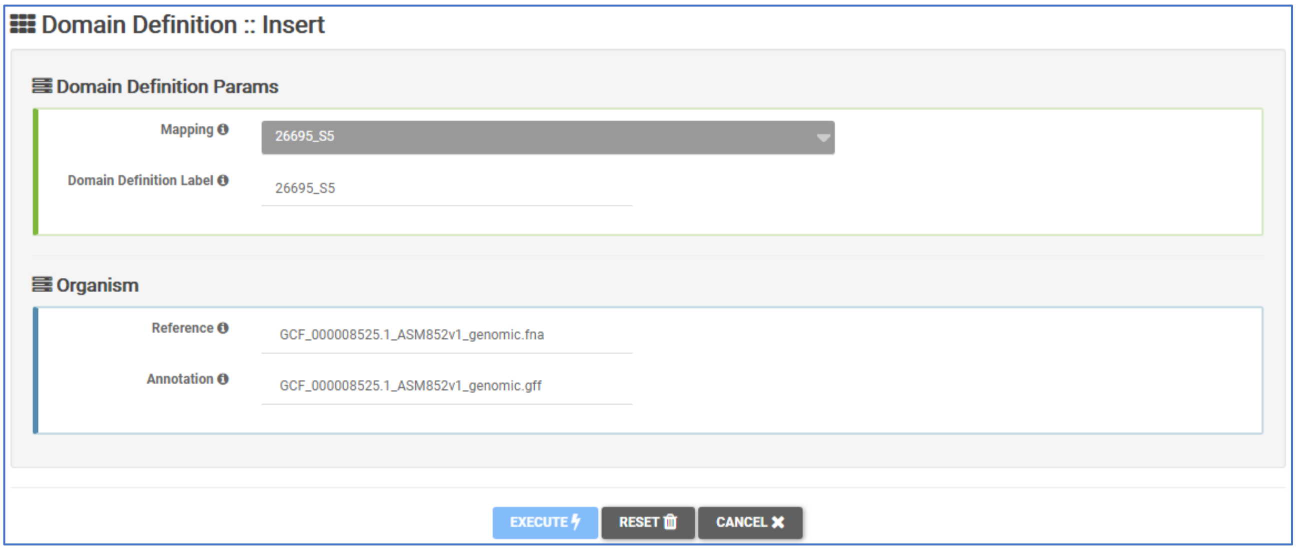

Domain Definition List shows the running domain definitions

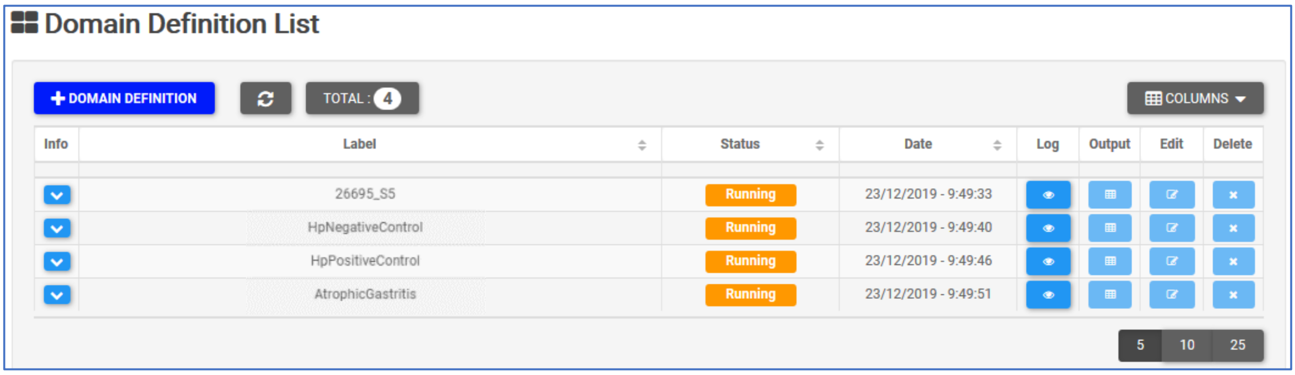

Info – Drop-down menu with information of Mapping input file**.**

Label – Sample label.

Status – When the execution ends successfully, the button turns green, otherwise, it turns red.

Date – Day and time of analysis execution

Log – Button that hides/opens a box with execution log file. When the execution is running, the log shows the possibility to stop it:

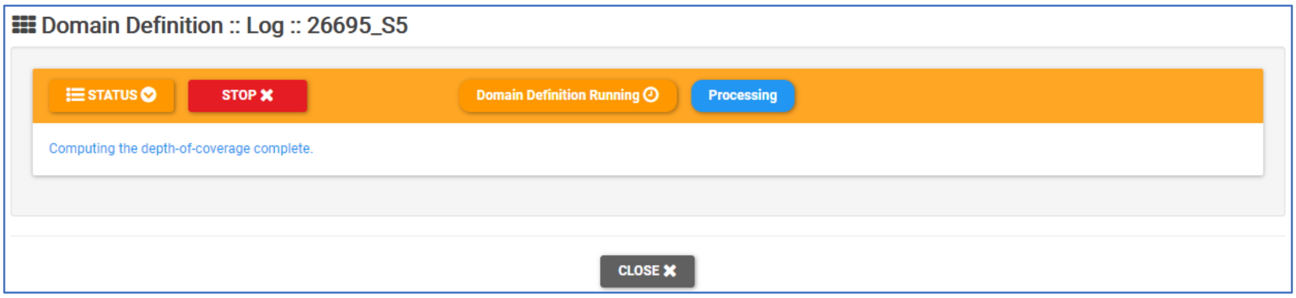

When the process has finished, the log shows summary information:

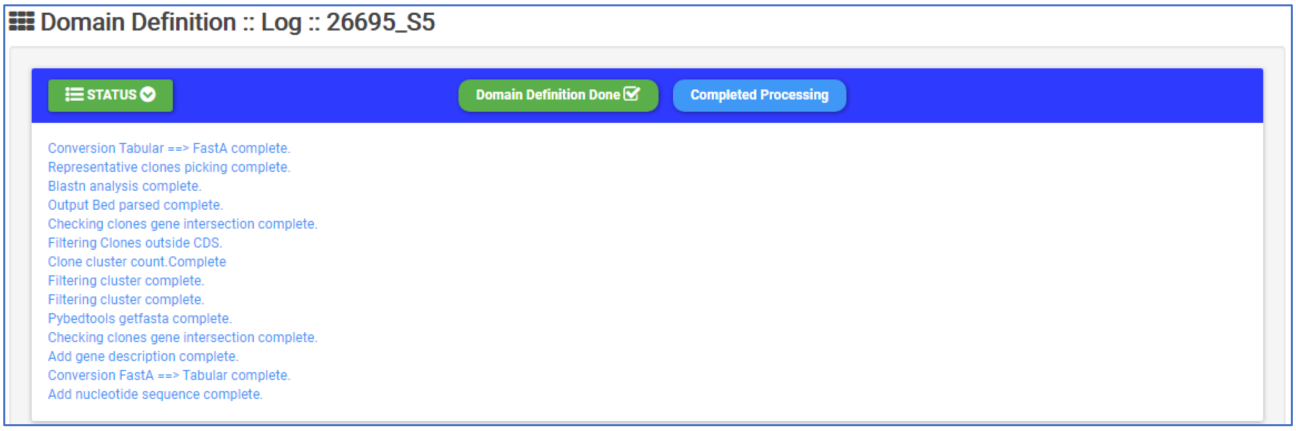

Output – Hides/opens panel with output preview

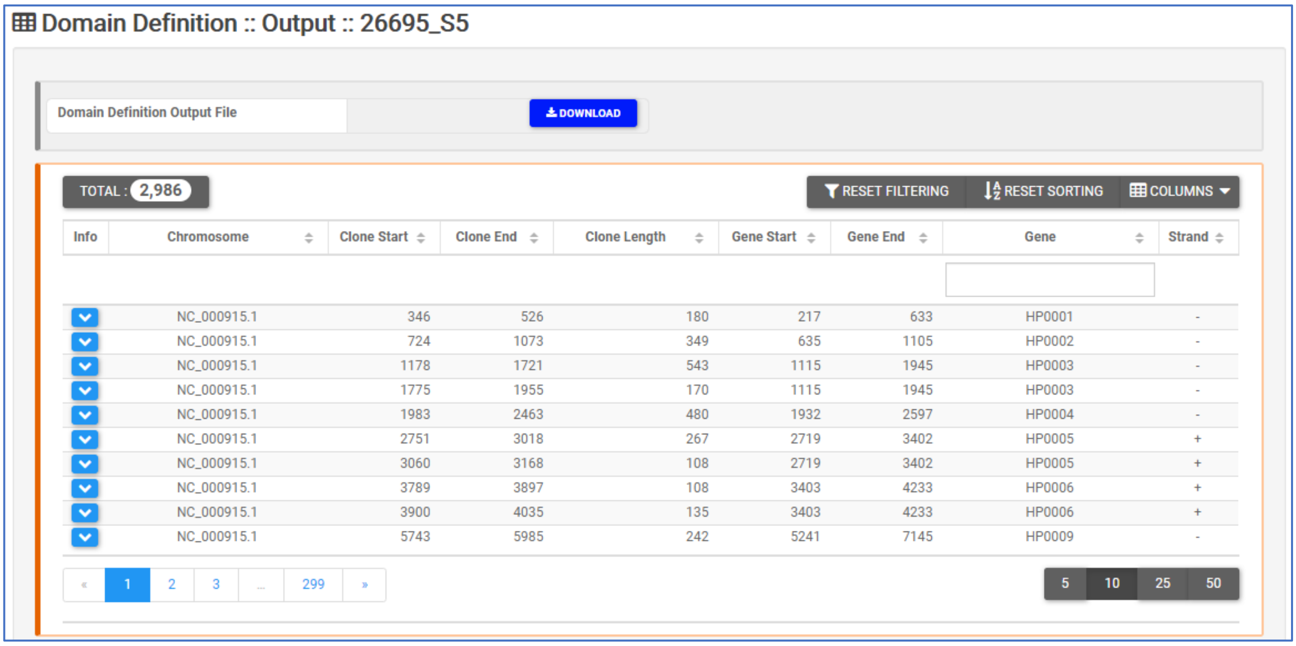

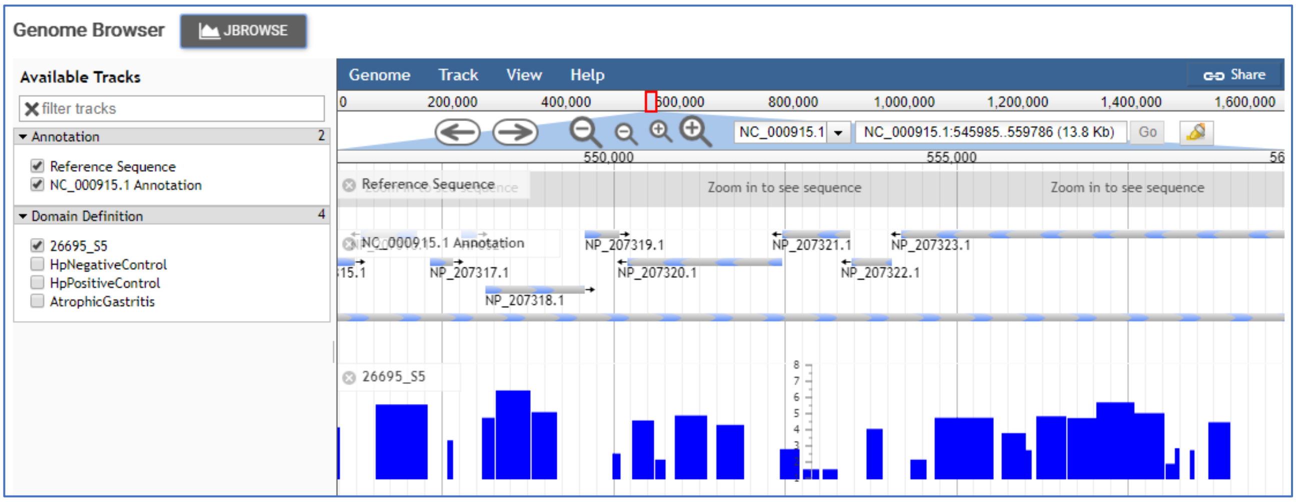

Domain Enrichment takes as input the Genomic and Target output of Domain Definition step. Enrich domains for each defined domain by selecting the Genomic and the target domains. In the current example, select:

- 26695_S5 and NegativeControl
- H26695_S5 and PositiveControl
- 26695_S5 and AtrophicGastritis

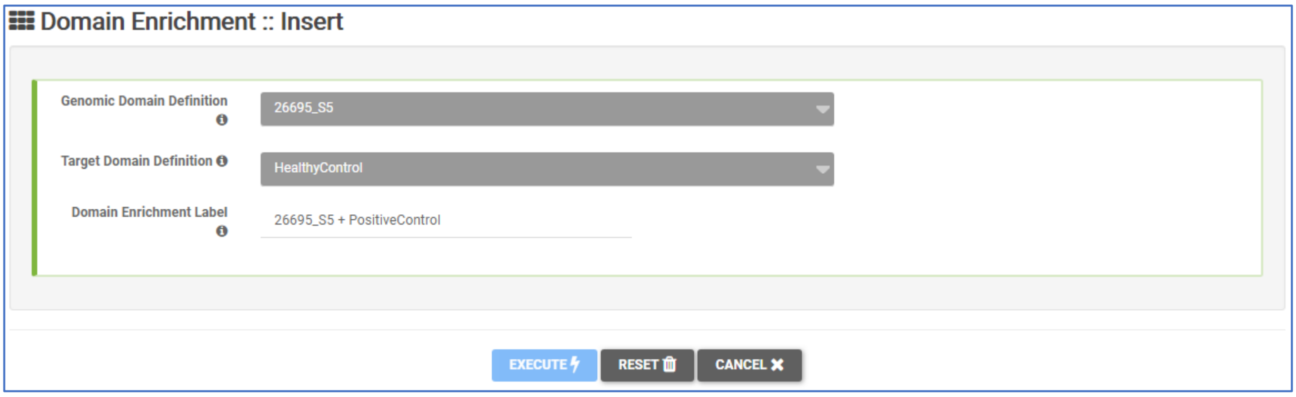

Domain Enrichment List

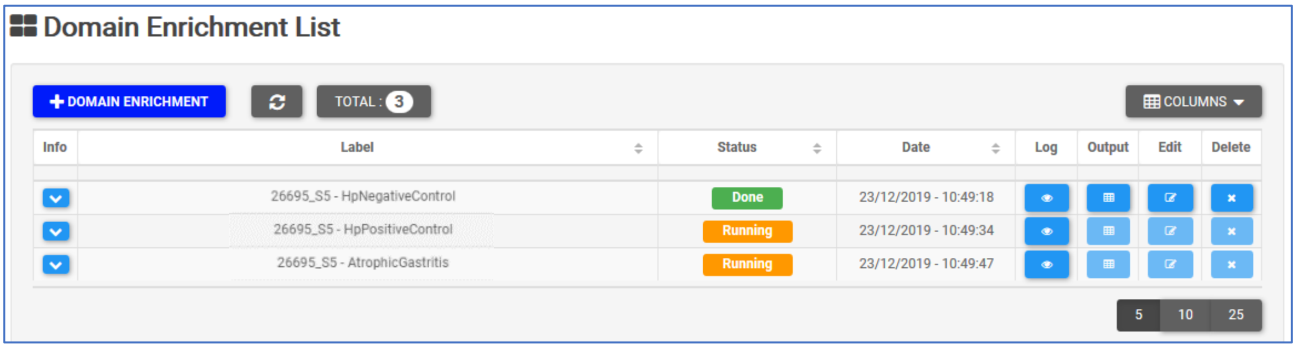

Info – Drop-down menu with information of Domain Definition input file**.**

Label – Sample label.

Status – When the execution ends successfully, the button turns green, otherwise, it turns red.

Date – Day and time of analysis execution

Log – Button that hides/opens a box with execution log file. When the execution is running, the log shows the possibility to stop it, otherwise the log shows summary information:

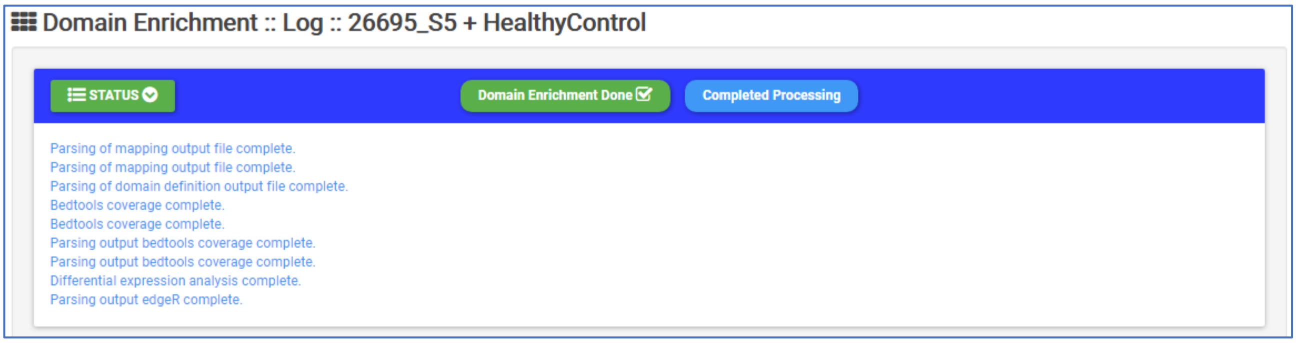

Output – Hides/opens panel with output preview

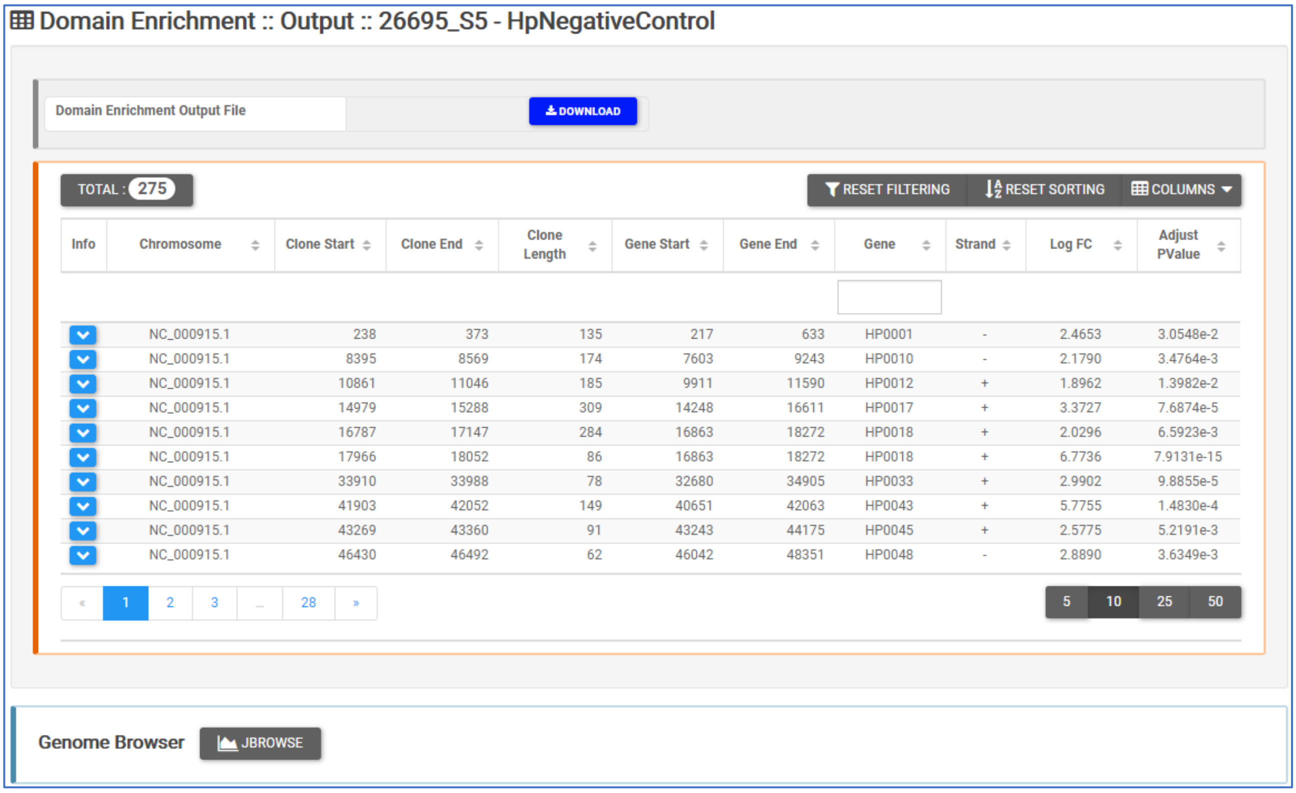

Info	Chromosome	Clone Start	Clone End	Clone Length	Gene Start	Gene End	Gene	Strand	Log FC	Adjust PValue
	NC_000915.1	238	373	135	217	633	HP0001	-	2.4653	3.0548e-2
	NC_000915.1	8395	8569	174	7603	9243	HP0010	-	2.1790	3.4764e-3
	NC_000915.1	10861	11046	185	9911	11590	HP0012	+	1.8962	1.3982e-2
	NC_000915.1	14979	15288	309	14248	16611	HP0017	+	3.3727	7.6874e-5
	NC_000915.1	16787	17147	284	16863	18272	HP0018	+	2.0296	6.5923e-3
	NC_000915.1	17966	18052	86	16863	18272	HP0018	+	6.7736	7.9131e-15
	NC_000915.1	33910	33988	78	32680	34905	HP0033	+	2.9902	9.8855e-5
	NC_000915.1	41903	42052	149	40651	42063	HP0043	+	5.7755	1.4830e-4
	NC_000915.1	43269	43360	91	43243	44175	HP0045	+	2.5775	5.2191e-3
	NC_000915.1	46430	46492	62	46042	48351	HP0048	-	2.8890	3.6349e-3

Domain Subtraction takes as input two differentially enriched epitopes/domains lists, one defined as Control Domain Enrichment and one defined as Selection Domain Enrichment. In this example, select the enrichments defined by the couples:

- 26695_S5 - HpPositiveControl
- 26695_S5 - AtrophicGastritis

then

- 26695_S5 – HpNegativeControl
- 26695_S5 – HpPositiveControl

and

- 26695_S5 – HpNegativeControl
- 26695_S5 – AtrophicGastritis

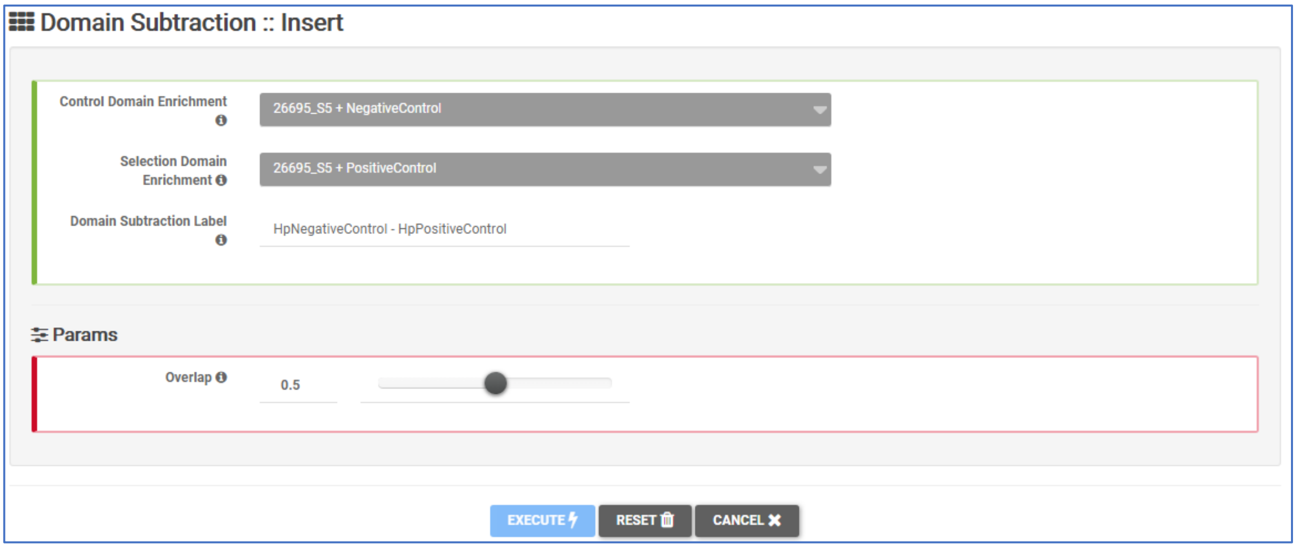

Domain subtraction list:

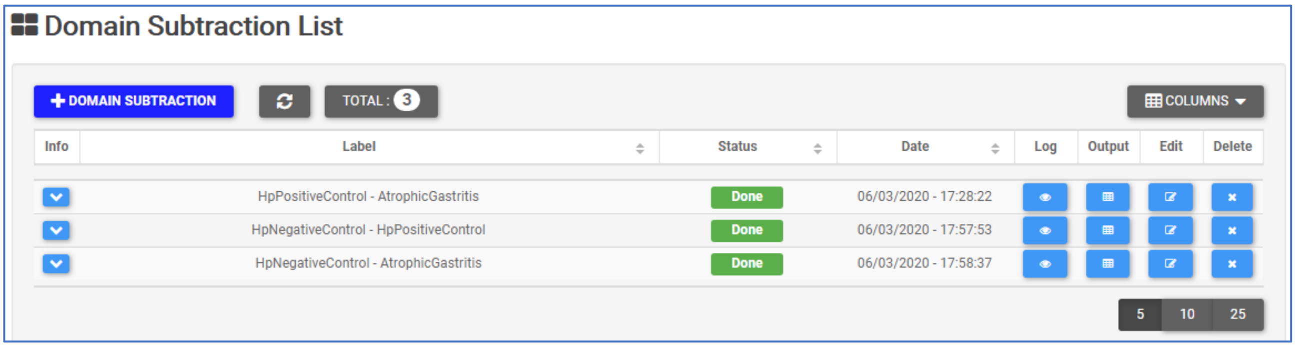

Info – Drop-down menu with information of Domain Enrichment input file**.**

Label – Sample label.

Status – When the execution ends successfully, the button turns green, otherwise, it turns red.

Date – Day and time of analysis execution

Log – Button that hides/opens a box with execution log file. When the execution is running, the log shows the possibility to stop it, otherwise the log shows summary information:

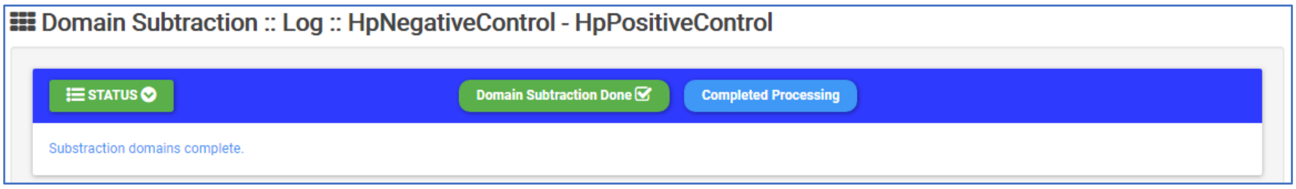

Output – Hides/opens panel with output preview

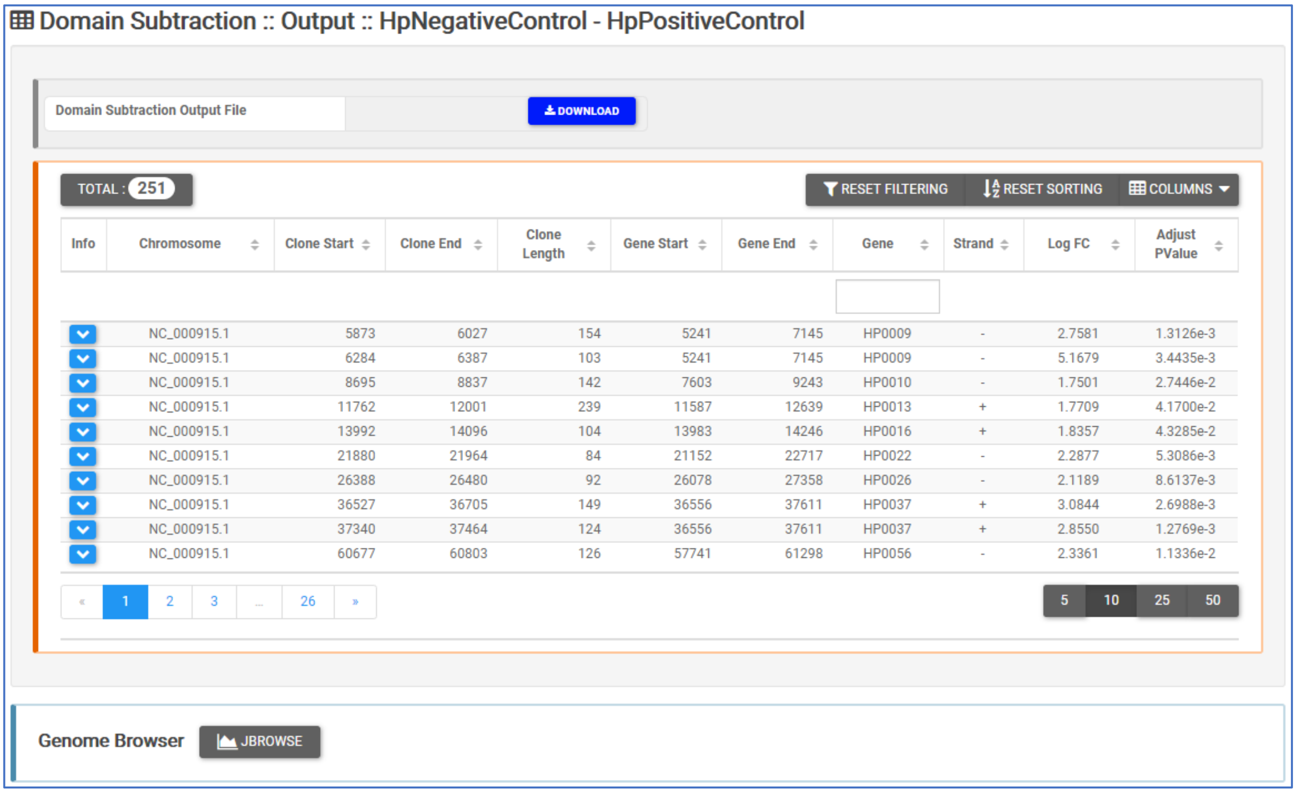

Info	Chromosome	Clone Start	Clone End	Clone Length	Gene Start	Gene End	Gene	Strand	Log FC	Adjust PValue
	NC_000915.1	5873	6027	154	5241	7145	HP0009	-	2.7581	1.3126e-3
	NC_000915.1	6284	6387	103	5241	7145	HP0009	-	5.1679	3.4435e-3
	NC_000915.1	8695	8837	142	7603	9243	HP0010	-	1.7501	2.7446e-2
	NC_000915.1	11762	12001	239	11587	12639	HP0013	+	1.7709	4.1700e-2
	NC_000915.1	13992	14096	104	13983	14246	HP0016	+	1.8357	4.3285e-2
	NC_000915.1	21880	21964	84	21152	22717	HP0022	-	2.2877	5.3086e-3
	NC_000915.1	26388	26480	92	26078	27358	HP0026	-	2.1189	8.6137e-3
	NC_000915.1	36527	36705	149	36556	37611	HP0037	+	3.0844	2.6988e-3
	NC_000915.1	37340	37464	124	36556	37611	HP0037	+	2.8550	1.2769e-3
	NC_000915.1	60677	60803	126	57741	61298	HP0056	-	2.3361	1.1336e-2

« 1 2 3 ... 26 » 5 10 25 50

Domain Intersection takes as input two differentially enriched epitopes/domains lists output of Domain Definition step. Domain Intersection allows one to screen for overlaps between two sets of epitopes/domains lists. Select both the subtractions previously executed and press **EXECUTE**

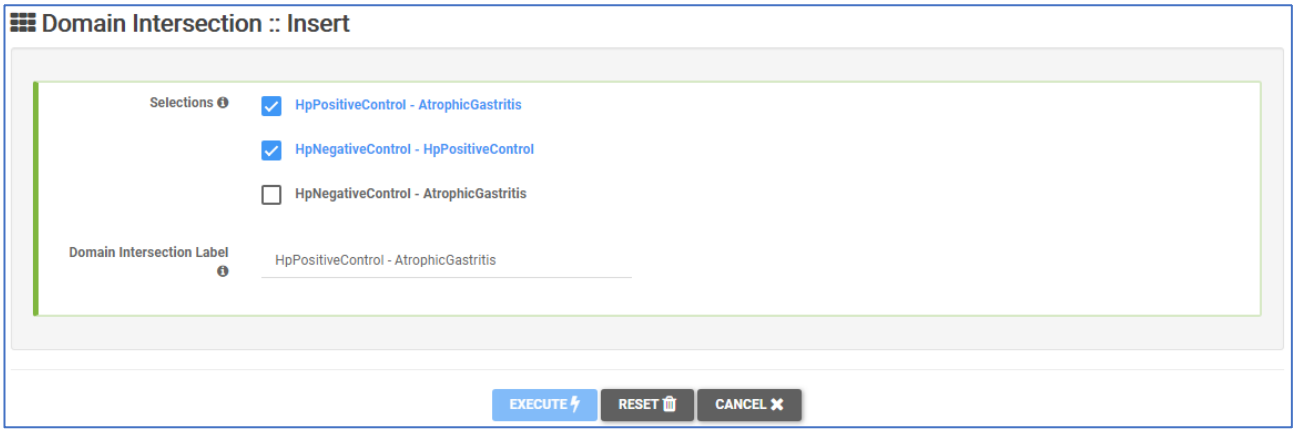

Domain intersection list:

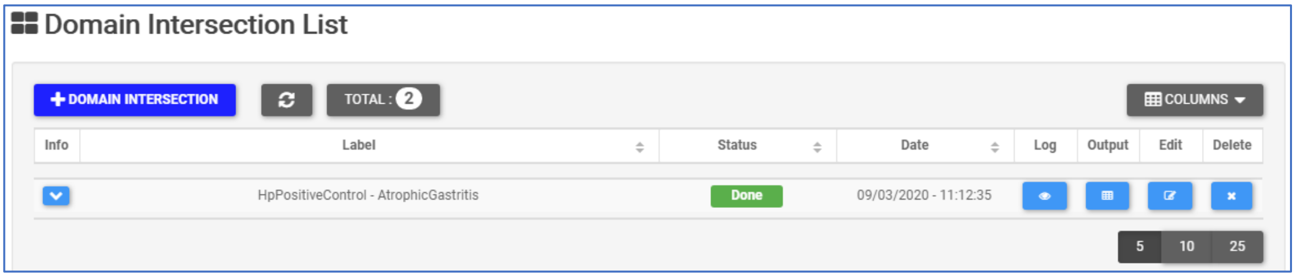

Info – Drop-down menu with information of Domain Enrichment input file**.**

Label – Sample label.

Status – When the execution ends successfully, the button turns green, otherwise, it turns red.

Date – Day and time of analysis execution

Log – Button that hides/opens a box with execution log file. When the execution is running, the log shows the possibility to stop it, otherwise the log shows summary information:

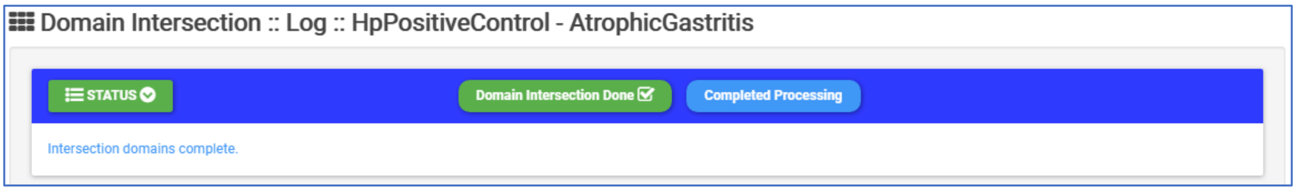

Output – Hides/opens panel with output preview

Domain Intersection :: Output :: HpNegativeControl - HpPositiveControl - AtrophicGastritis

LEGEND

- [A] HpPositiveControl - AtrophicGastritis
- [B] HpNegativeControl - HpPositiveControl
- [C] HpNegativeControl - AtrophicGastritis

Domain Intersection Output Files — DOWNLOAD

Domain Intersection Graph File — PNG | SVG

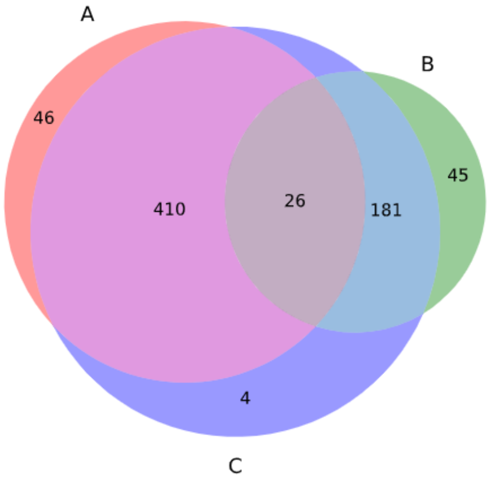

- A - (B ∪ C)
- B - (A ∪ C)
- C - (A ∪ B)
- A ∩ B
- A ∩ C
- B ∩ C
- A ∩ B ∩ C

Domain Intersection Output File — DOWNLOAD

TOTAL : 26 | RESET FILTERING | RESET SORTING | COLUMNS

Info	Chromosome	Clone Start	Clone End	Clone Length	Gene Start	Gene End	Gene	Strand	Log FC	Adjust PValue
	NC_000915.1	8695	9048	353	7603	9243	HP0010	-	2.4847	1.0268e-3
	NC_000915.1	65698	65905	207	64016	66457	HP0060	+	2.4895	1.8192e-3
	NC_000915.1	120280	120498	218	119980	120675	HP0112	+	5.2941	5.3429e-8
	NC_000915.1	196397	196650	253	195596	197104	HP0190	-	2.9338	1.5543e-4
	NC_000915.1	198654	198889	235	197856	200000	HP0192	-	2.1820	3.9586e-3
	NC_000915.1	206920	207090	170	206915	207910	HP0202	+	2.3630	3.5821e-3

Results

The summary of all the analysis performed is showed in the results page

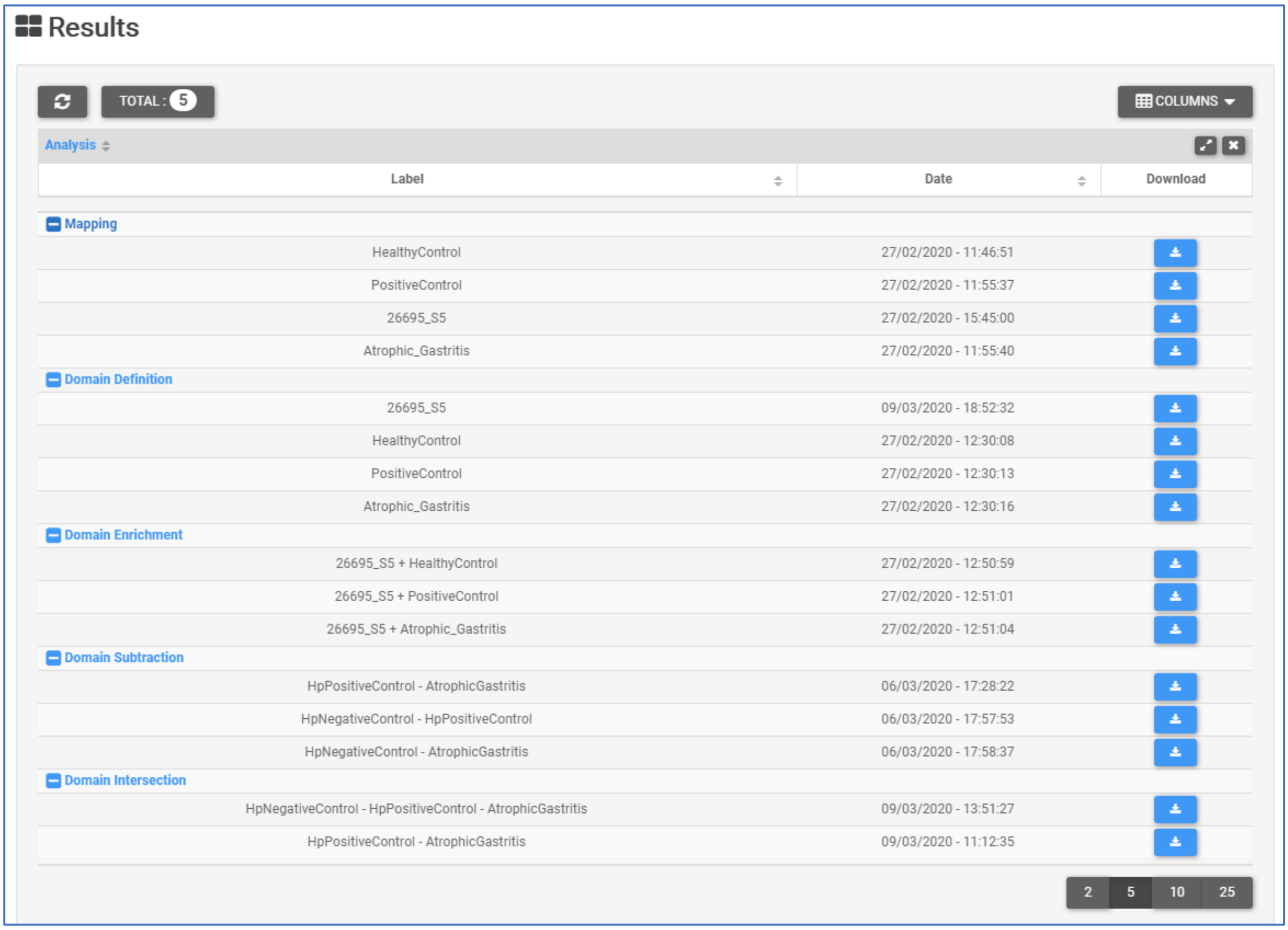

Results

TOTAL : 5 | COLUMNS

Label	Date	Download
**Mapping**		
HealthyControl	27/02/2020 - 11:46:51	
PositiveControl	27/02/2020 - 11:55:37	
26695_S5	27/02/2020 - 15:45:00	
Atrophic_Gastritis	27/02/2020 - 11:55:40	
**Domain Definition**		
26695_S5	09/03/2020 - 18:52:32	
HealthyControl	27/02/2020 - 12:30:08	
PositiveControl	27/02/2020 - 12:30:13	
Atrophic_Gastritis	27/02/2020 - 12:30:16	
**Domain Enrichment**		
26695_S5 + HealthyControl	27/02/2020 - 12:50:59	
26695_S5 + PositiveControl	27/02/2020 - 12:51:01	
26695_S5 + Atrophic_Gastritis	27/02/2020 - 12:51:04	
**Domain Subtraction**		
HpPositiveControl - AtrophicGastritis	06/03/2020 - 17:28:22	
HpNegativeControl - HpPositiveControl	06/03/2020 - 17:57:53	
HpNegativeControl - AtrophicGastritis	06/03/2020 - 17:58:37	
**Domain Intersection**		
HpNegativeControl - HpPositiveControl - AtrophicGastritis	09/03/2020 - 13:51:27	
HpPositiveControl - AtrophicGastritis	09/03/2020 - 11:12:35	

2 5 10 25

And a genome browser with the results aligned to the reference genome

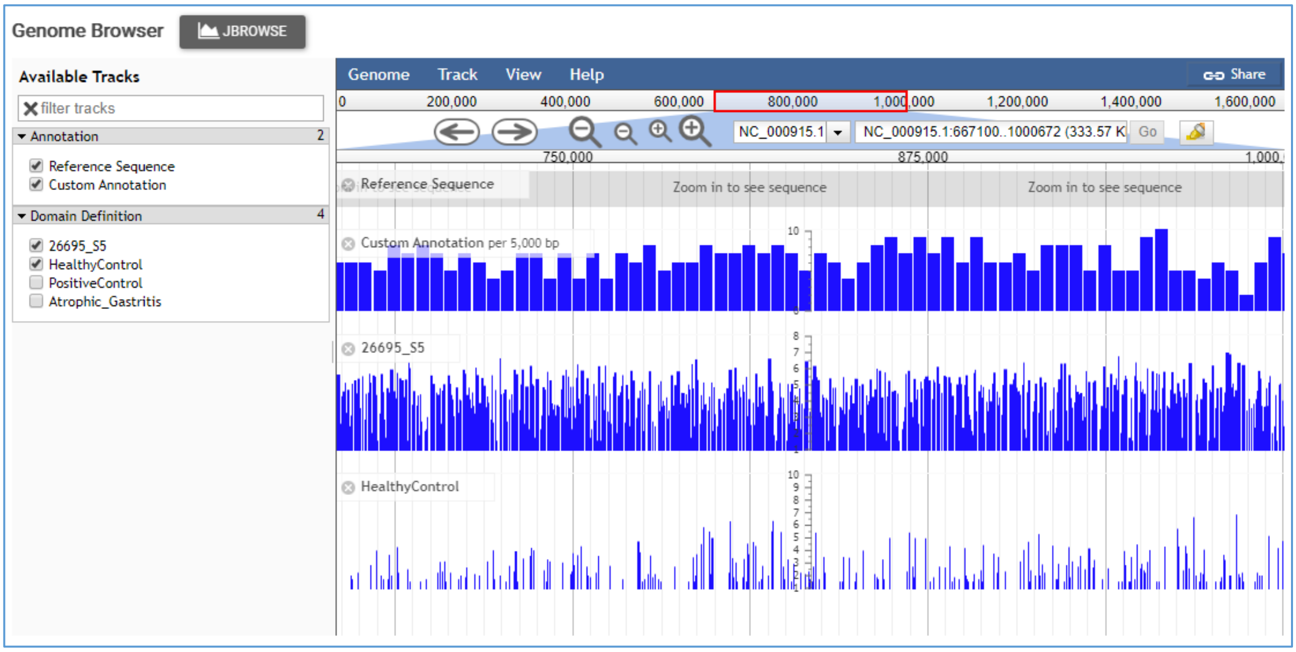

Eukaryote - Tutorial RnaBindProt

This introductory section provides an overview of **Eukaryote** pipeline drafting and design. The vertical gray rectangles correspond to the website sections.

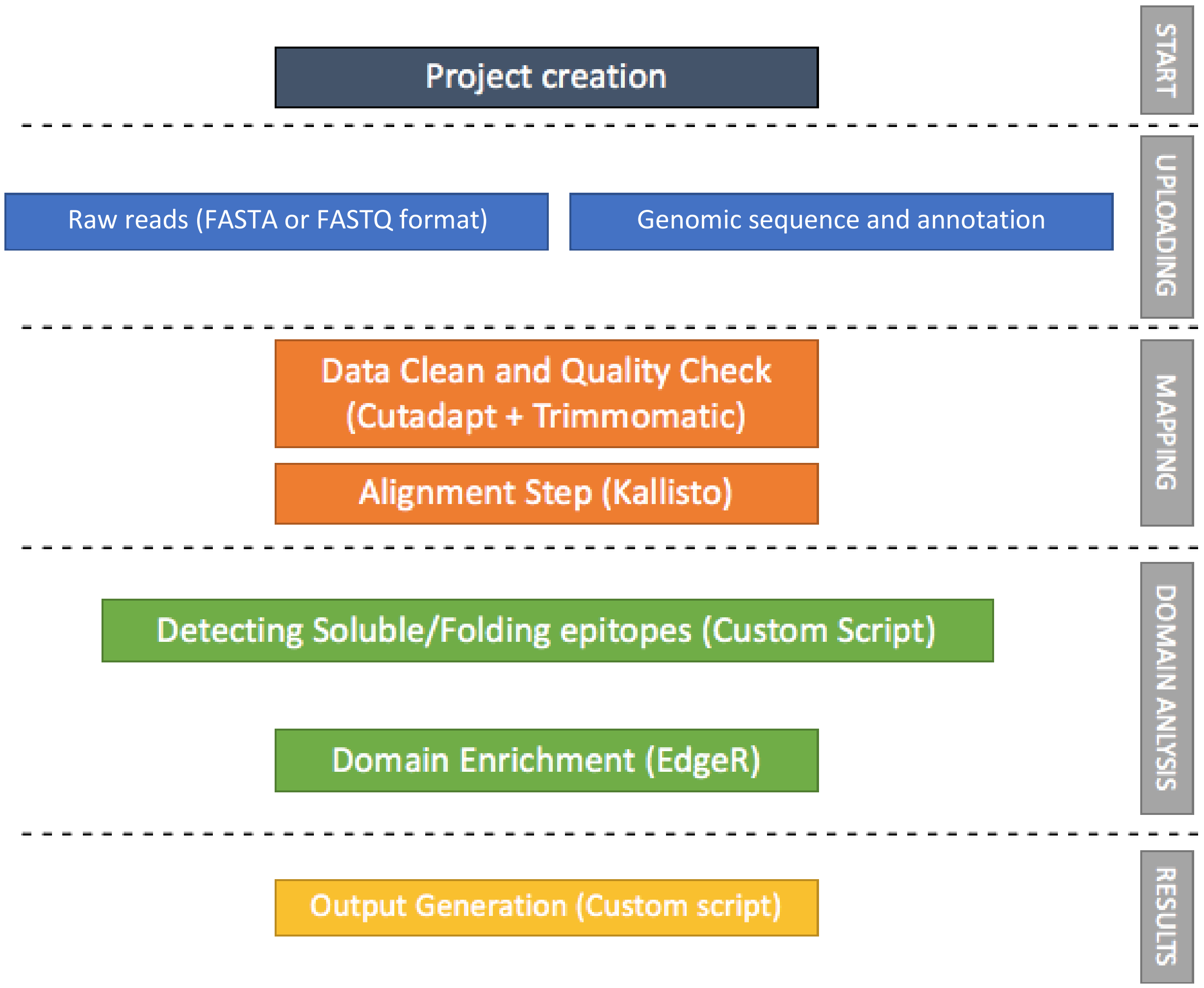

This document shows how to perform all the steps of an example analysis with the data provided for Eukaryote pipeline. For further information about the forms and the interpretation of the results, refer to the User Guide.

Input Files: dataset Eukaryote_RnaBindProt.zip

Download a zip folder containing the raw datasets from this link (105 MB).

Mandatory inputs for **InteractomeSeq - Eukaryote RnaBindProt** execution are:

- Raw Data files, FASTA or FASTQ format for query reads are allowed in the input, therefore the web interface additionally allows the submission of compressed files (gz format) to reduce the time of data upload (DataSets).
  - Delta5Uchl1ssDNA.fastq (86 MB)
  - Delta5Uchl1tRNA.fastq (7 MB)
  - invSINEB2ssDNA.fastq (4 MB)
  - invSINEB2tRNA.fastq (4 MB)
  - NotSelected.fastq (4 MB)

InteractomeSeq requires at least two datasets. The input datasets must be generated with the same sequencing platform.

Time and memory usage for this tutorial

Analysis Step	File	Excution time	Output size
Mapping	Delta5Uchl1ssDNA	~ 17m	133 MB
Mapping	Delta5Uchl1tRNA	~ 4m	12 MB
Mapping	invSINEB2ssDNA	~ 4m	7.7 MB
Mapping	invSINEB2tRNA	~ 4m	7.0 MB
Mapping	NotSelected	~ 3m	6.3 MB
Domain Definition	Delta5Uchl1ssDNA	~ 23m	2.4 MB
Domain Definition	Delta5Uchl1tRNA	~ 4m	736 KB
Domain Definition	invSINEB2ssDNA	~ 4m	652 KB
Domain Definition	invSINEB2tRNA	~ 4m	686 KB
Domain Definition	NotSelected	~ 4m	1.5 MB
Domain Enrichment	Delta5Uchl1ssDNA - Delta5Uchl1tRNA	< 5s	193 KB
Domain Enrichment	invSINEB2ssDNA - invSINEB2tRNA	< 5s	226 KB

Create a project

Click the button **START** to create a new project. Give the project a name (mandatory), a description (optional) and an email address (to receive messages during the execution. Select **EUKARYOTE** as project type and **SAVE**.

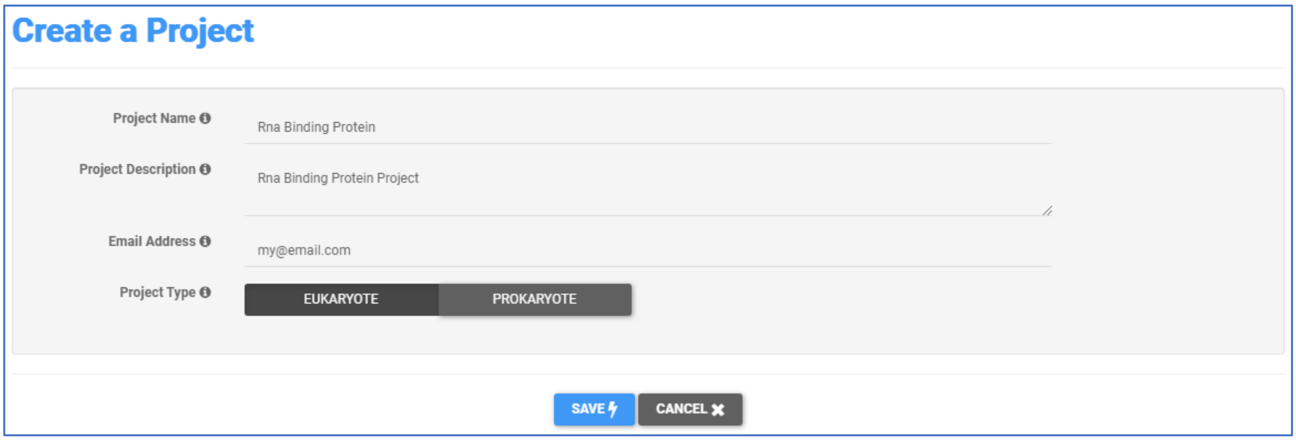

The project now has an ID that is a link, active for 15 days. Save this link if you didn't provide an email address (the link will be sent via email).

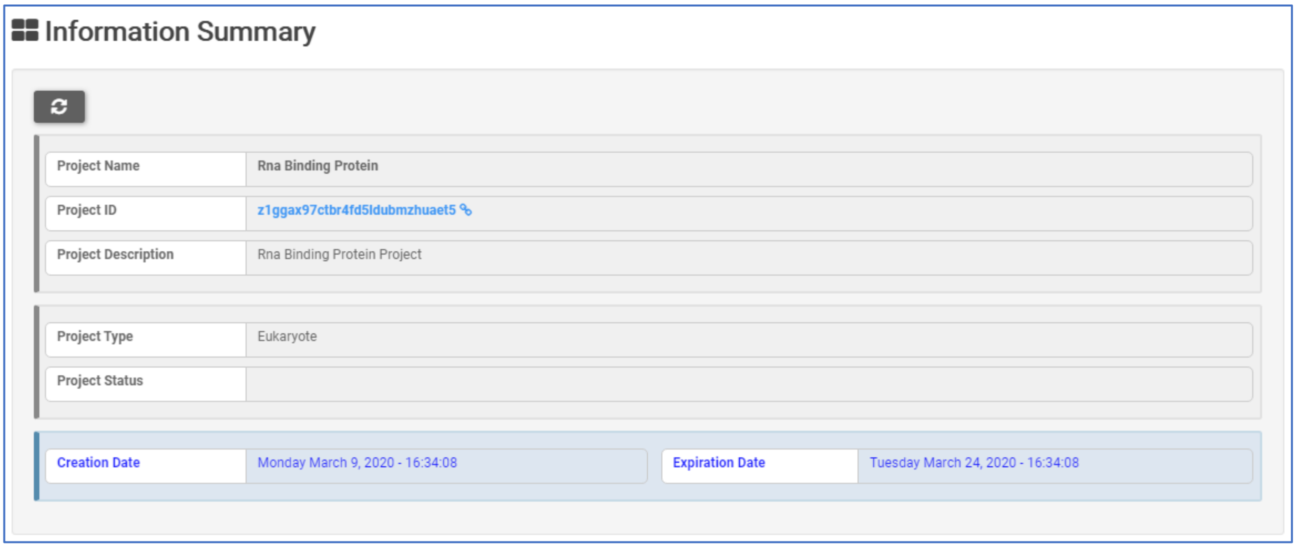

Uploading

Clik on **UPLOADING** to upload the FASTQ datasets.

Select the annotation to use for the dataset mapping. In this case, select **HOMO SAPIENS**. Pre-loaded nucleotide sequences of all protein-coding transcripts and annotation file are available for Homo Sapiens and Mus Musculus genome. The genome assembly version for Homo Sapiens is the GRCh38 downloaded from NCBI and the annotation was downloaded from GenCode consortium https://www.gencodegenes.org/human/. The genome assembly version for Mus Musculus is the GRCm38 downloaded from NCBI and the annotation was downloaded from GenCode consortium https://www.gencodegenes.org/mouse/.

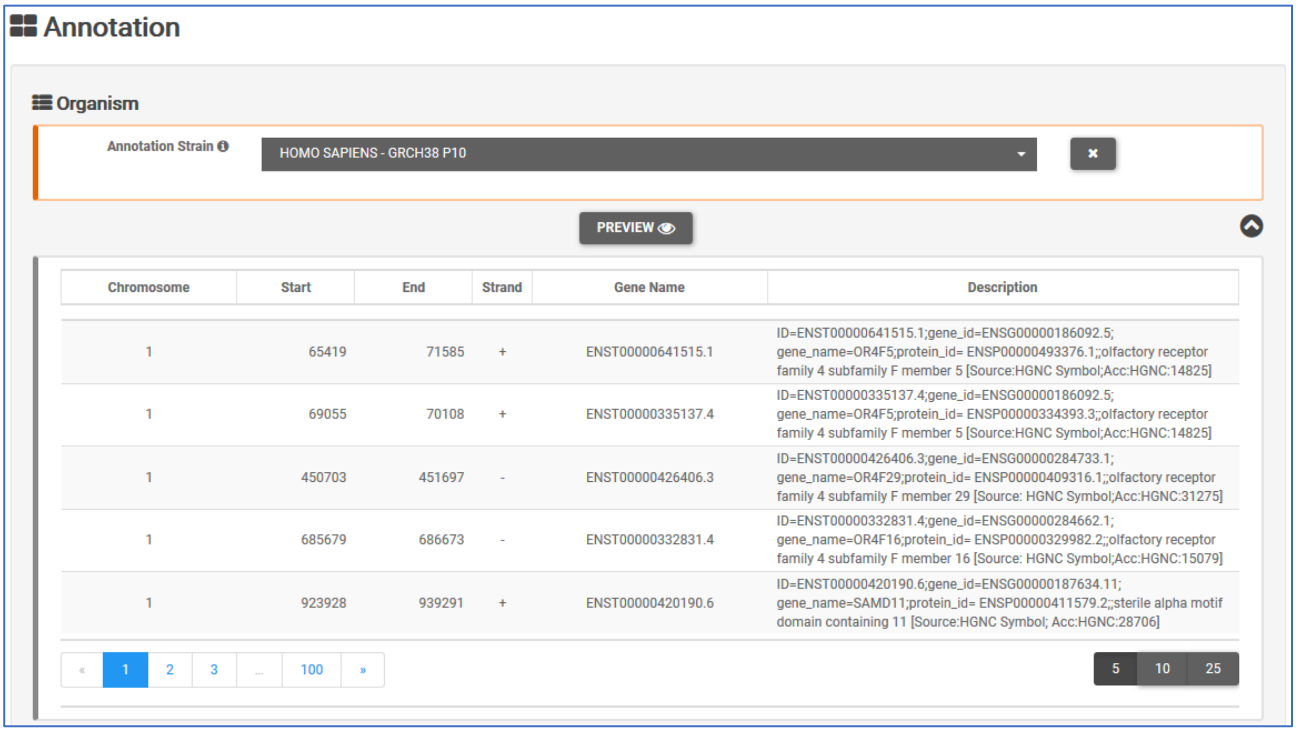

Chromosome	Start	End	Strand	Gene Name	Description
1	65419	71585	+	ENST00000641515.1	ID=ENST00000641515.1;gene_id=ENSG00000186092.5; gene_name=OR4F5;protein_id= ENSP00000493376.1;;olfactory receptor family 4 subfamily F member 5 [Source:HGNC Symbol;Acc:HGNC:14825]
1	69055	70108	+	ENST00000335137.4	ID=ENST00000335137.4;gene_id=ENSG00000186092.5; gene_name=OR4F5;protein_id= ENSP00000334393.3;;olfactory receptor family 4 subfamily F member 5 [Source:HGNC Symbol;Acc:HGNC:14825]
1	450703	451697	-	ENST00000426406.3	ID=ENST00000426406.3;gene_id=ENSG00000284733.1; gene_name=OR4F29;protein_id= ENSP00000409316.1;;olfactory receptor family 4 subfamily F member 29 [Source: HGNC Symbol;Acc:HGNC:31275]
1	685679	686673	-	ENST00000332831.4	ID=ENST00000332831.4;gene_id=ENSG00000284662.1; gene_name=OR4F16;protein_id= ENSP00000329982.2;;olfactory receptor family 4 subfamily F member 16 [Source: HGNC Symbol;Acc:HGNC:15079]
1	923928	939291	+	ENST00000420190.6	ID=ENST00000420190.6;gene_id=ENSG00000187634.11; gene_name=SAMD11;protein_id= ENSP00000411579.2;;sterile alpha motif domain containing 11 [Source:HGNC Symbol; Acc:HGNC:28706]

In the **DataSets** section, click on **SELECT FILE** or drag&drop files to upload FASTQ datasets:

- Delta5Uchl1ssDNA.fastq.gz
- Delta5Uchl1tRNA.fastq.gz
- invSINEB2ssDNA.fastq.gz
- invSINEB2tRNA.fastq.gz
- NotSelected.fastq.gz

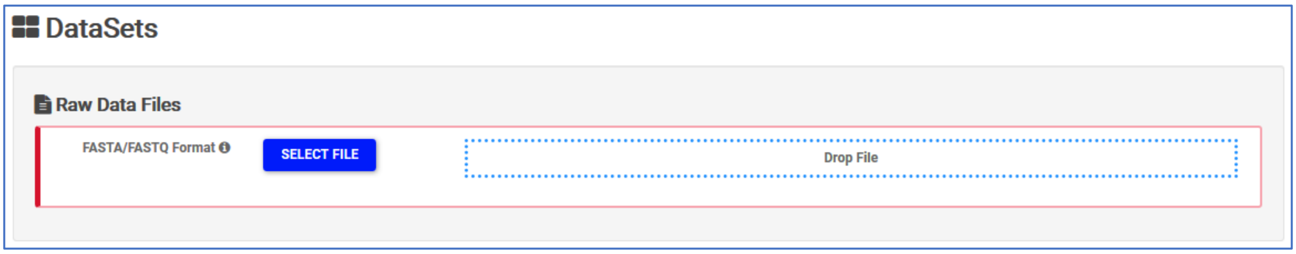

Dataset loading visualization:

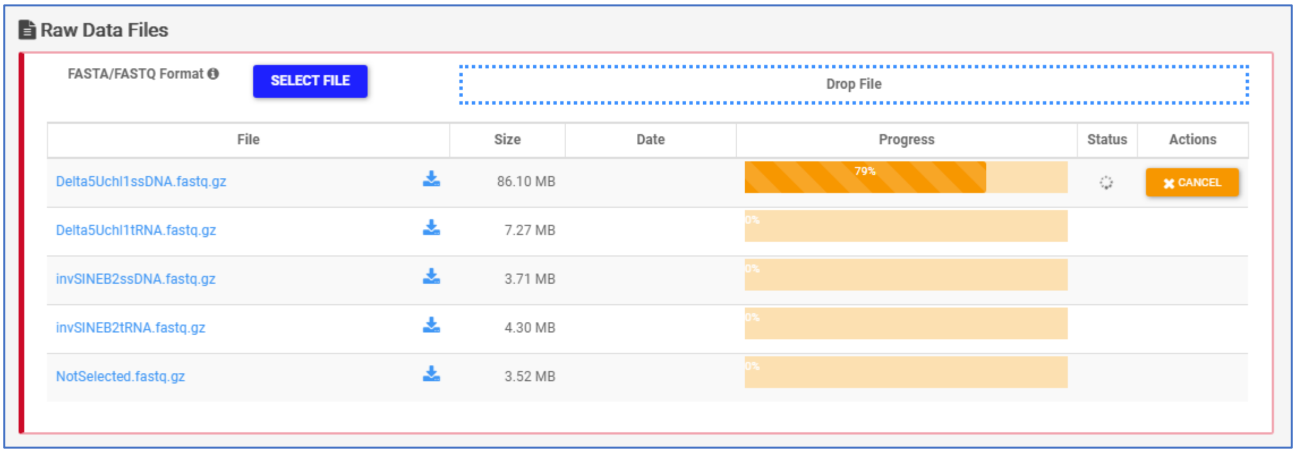

Mapping

In the **MAPPING** section, by clicking on the button **+ MAPPING,** 4 sub-sections will appear on the screen:

5. **Mapping Params.** Select single-end reads (in this example: NotSelected)

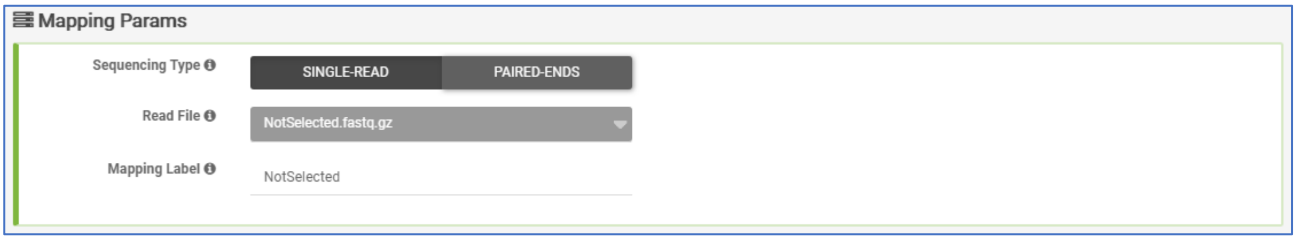

6. **Organism.** Preloaded FASTA file that will be used as reference to align the sequences (Homo Sapiens).

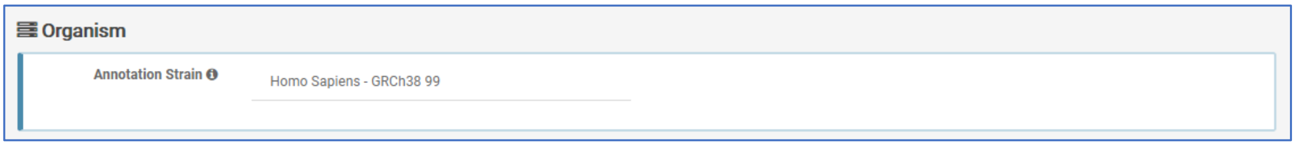

7. **Adapters.** Select **Custom Adapters** and insert:
   a. Forward Read 5' Adapter GCAGCAAGCGGCGCGCATGC
   b. Forward Read 3' Adapter GCGCTTCGTCAT

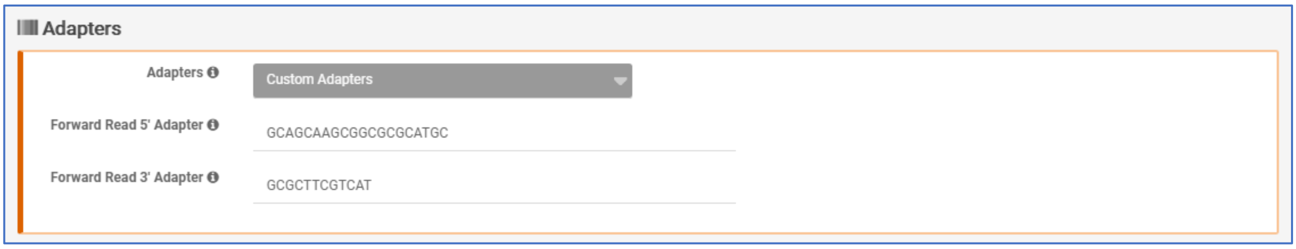

8. **Trimming Params.** Selection of minimum length of sequence and number of allowed mismatches, reads below this thresholds will be discarded. Use the default, and click **EXECUTE**

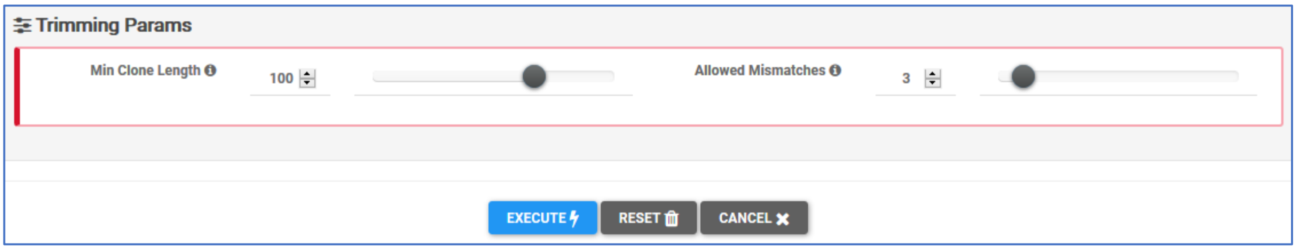

This mapping will appear in the list of running mappings.

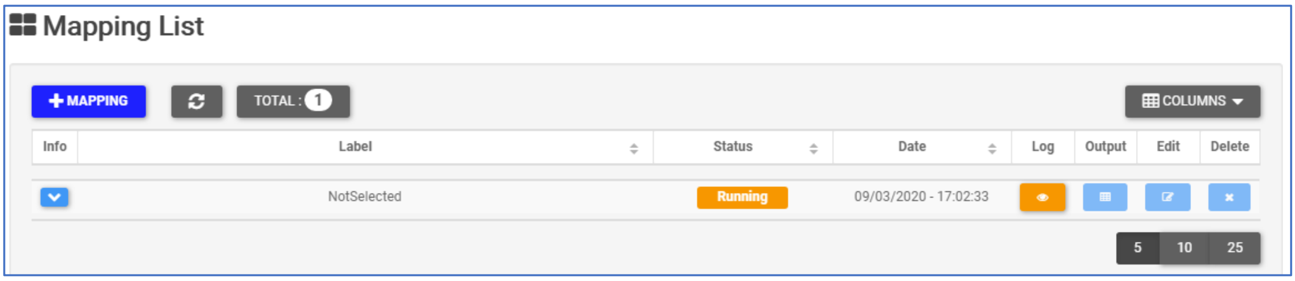

Info – Drop-down menu with information of Mapping input file**.**

Label – Sample label.

Status – When the execution ends successfully, the button turns green, otherwise, it turns red.

Date – Day and time of analysis execution

Log – Button that hides/opens a box with execution log file. When the execution is running, the log shows the possibility to stop it:

Mapping :: Log :: NotSelected

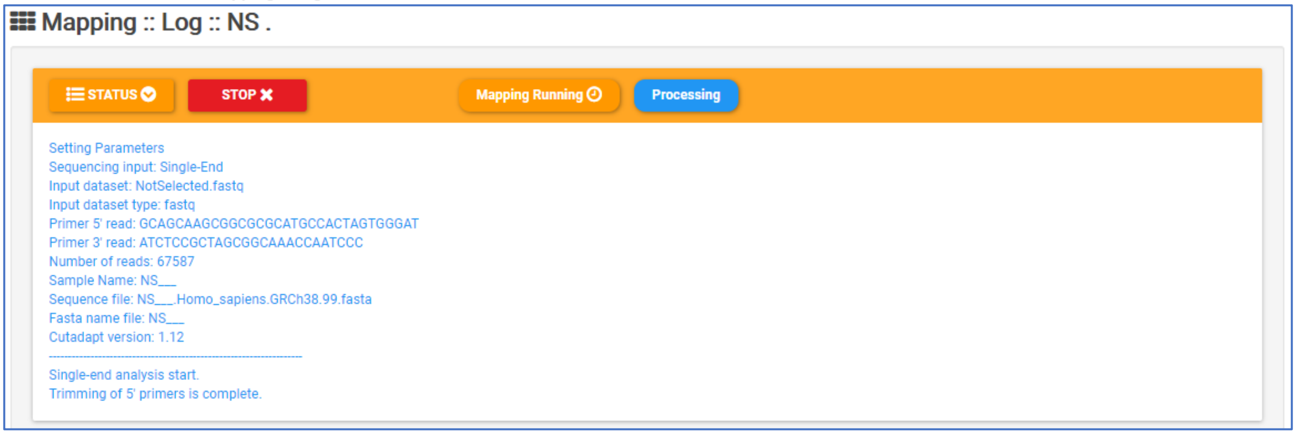

When the process has finished, the log shows summary information:

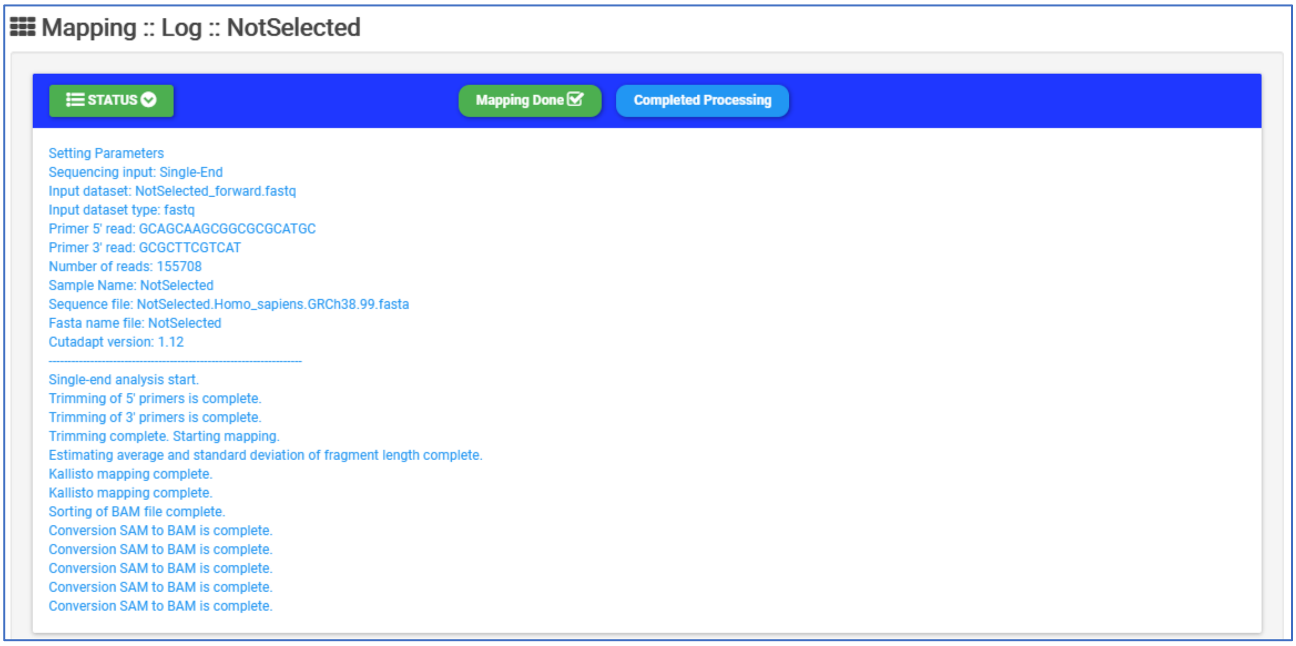

Output – Hides/opens panel with download button

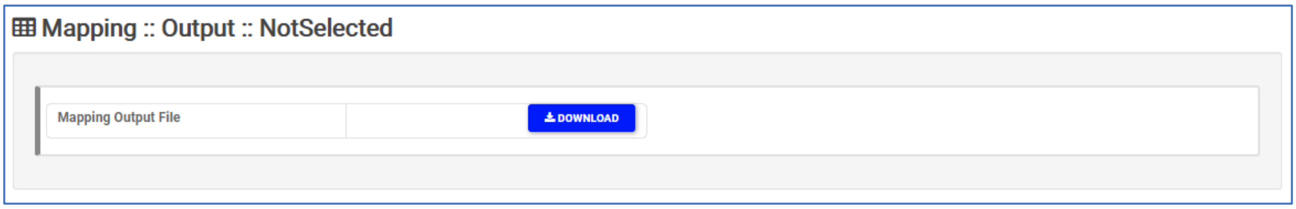

Here are the parameters for the other 4 mappings in this example:

4. Delta5Uchl1ssDNA

   SequencingType Single-Read

   Forward Read File Delta5Uchl1ssDNA.fastq.gz

   Annotation Strain Homo Sapiens - GRCh38 p10

   AdapterType Custom

   Forward Read 5' Adapter GCAGCAAGCGGCGCGCATGC

   Forward Read 3' Adapter GCGCTTCGTCAT

   Min Clone Length 100

   Allowed Mismatches 3

5. Delta5Uchl1tRNA

   SequencingType Single-Read

   Forward Read File Delta5Uchl1tRNA.fastq.gz

   Annotation Strain Homo Sapiens - GRCh38 p10

   AdapterType Custom

   Forward Read 5' Adapter GCAGCAAGCGGCGCGCATGC

   Forward Read 3' Adapter GCGCTTCGTCAT

   Min Clone Length 100

   Allowed Mismatches 3

6. invSINEB2ssDNA

   SequencingType Single-Read

   Forward Read File invSINEB2ssDNA.fastq.gz

   Annotation Strain Homo Sapiens - GRCh38 p10

   AdapterType Custom

   Forward Read 5' Adapter GCAGCAAGCGGCGCGCATGC

   Forward Read 3' Adapter GCGCTTCGTCAT

   Min Clone Length 100

   Allowed Mismatches 3

7. invSINEB2tRNA

   SequencingType Single-Read

   Forward Read File invSINEB2tRNA.fastq.gz

   Annotation Strain Homo Sapiens - GRCh38 p10

   AdapterType Custom

   Forward Read 5' Adapter GCAGCAAGCGGCGCGCATGC

   Forward Read 3' Adapter GCGCTTCGTCAT

   Min Clone Length 100

   Allowed Mismatches 3

This is the list of running mappings:

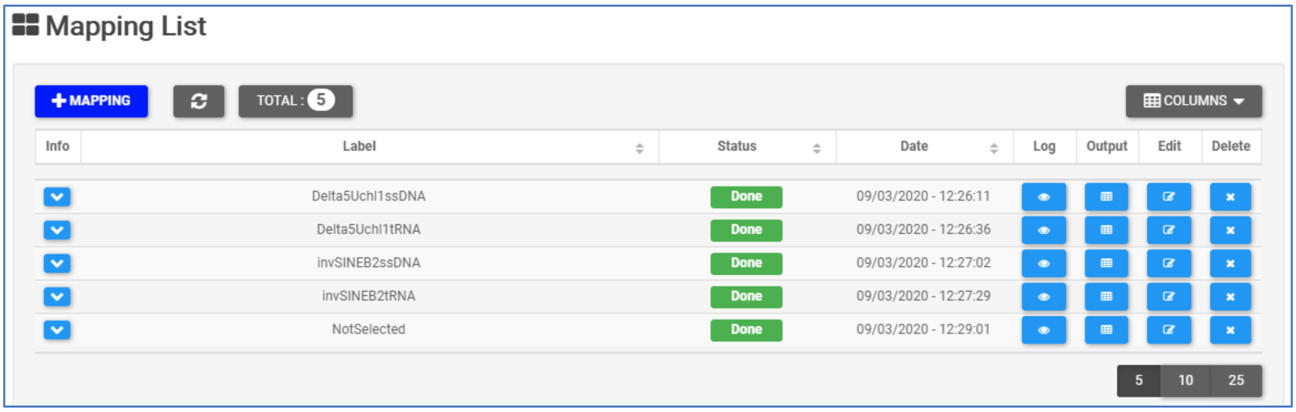

Domain Analysis

Domain analysis is composed by two sheets:

5. **Domain Definition**
6. **Domain Enrichment**

Domain Definition takes as input the mapping file previously generated. Define the domains for each of the 5 datasets uploaded and mapped by choosing them in the **Mapping** menu, then press **Execute**.

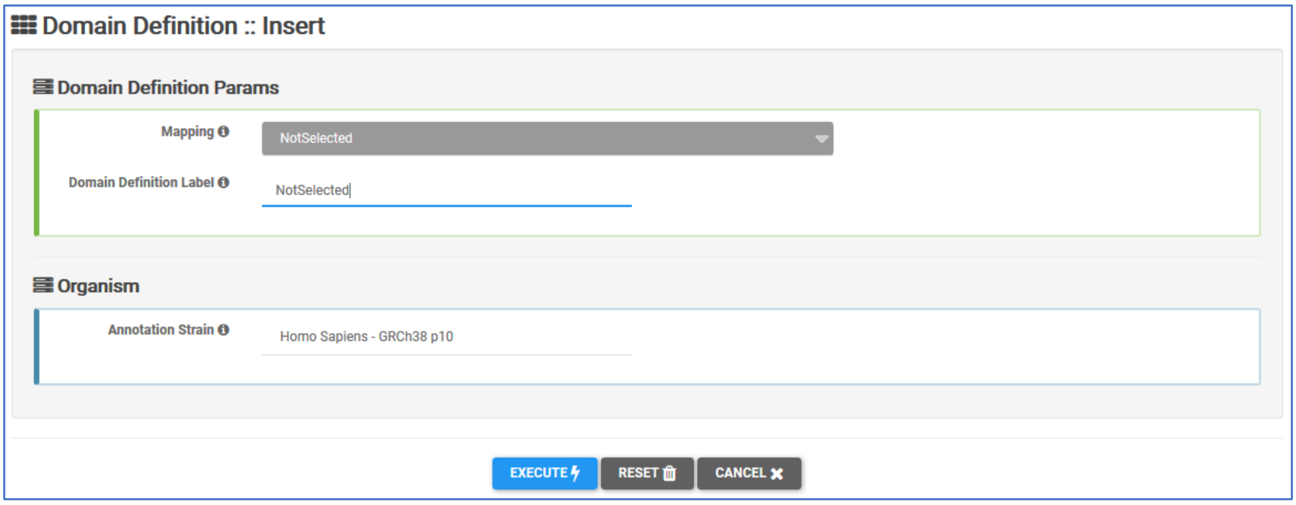

Domain Definition List shows the running domain definitions

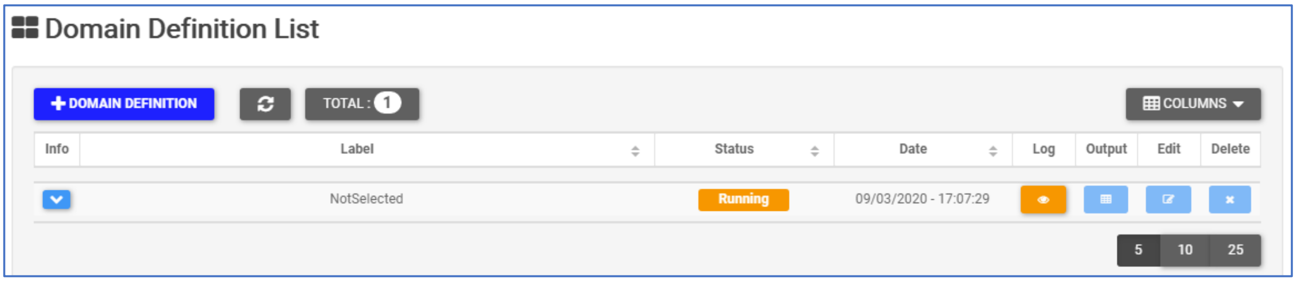

Info – Drop-down menu with information of Mapping input file**.**

Label – Sample label.

Status – When the execution ends successfully, the button turns green, otherwise, it turns red.

Date – Day and time of analysis execution

Log – Button that hides/opens a box with execution log file. When the execution is running, the log shows the possibility to stop it:

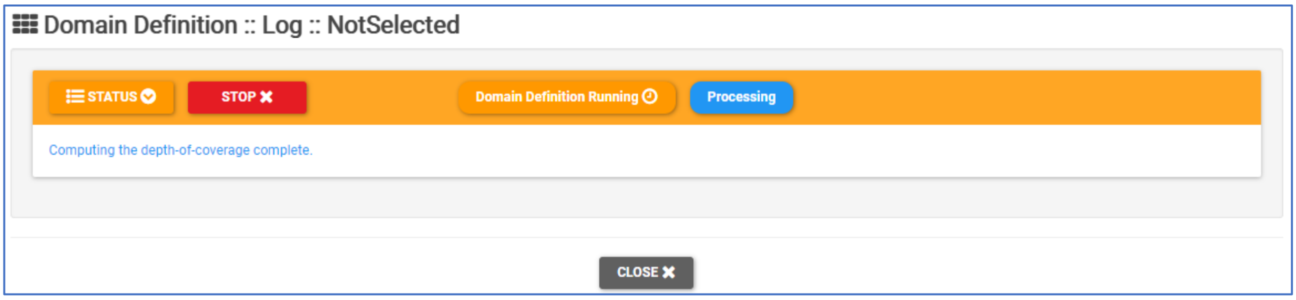

When the process has finished, the log shows summary information:

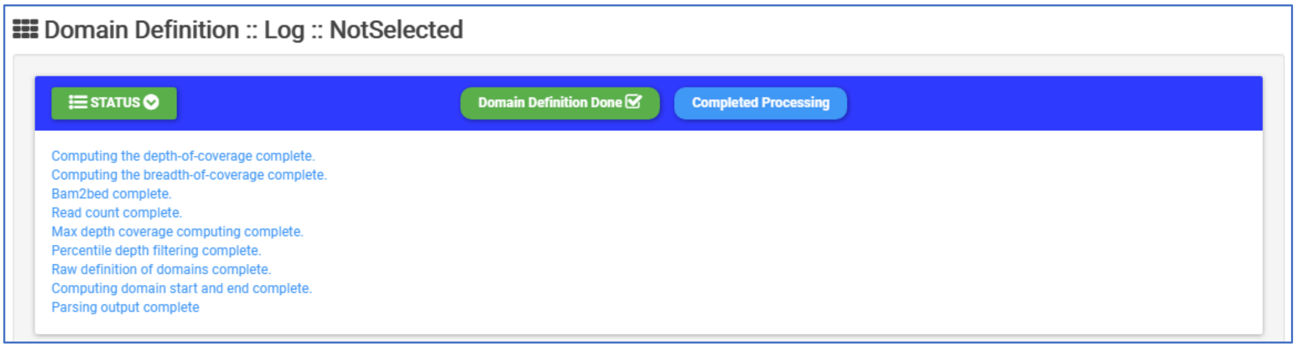

Output – Hides/opens panel with output preview

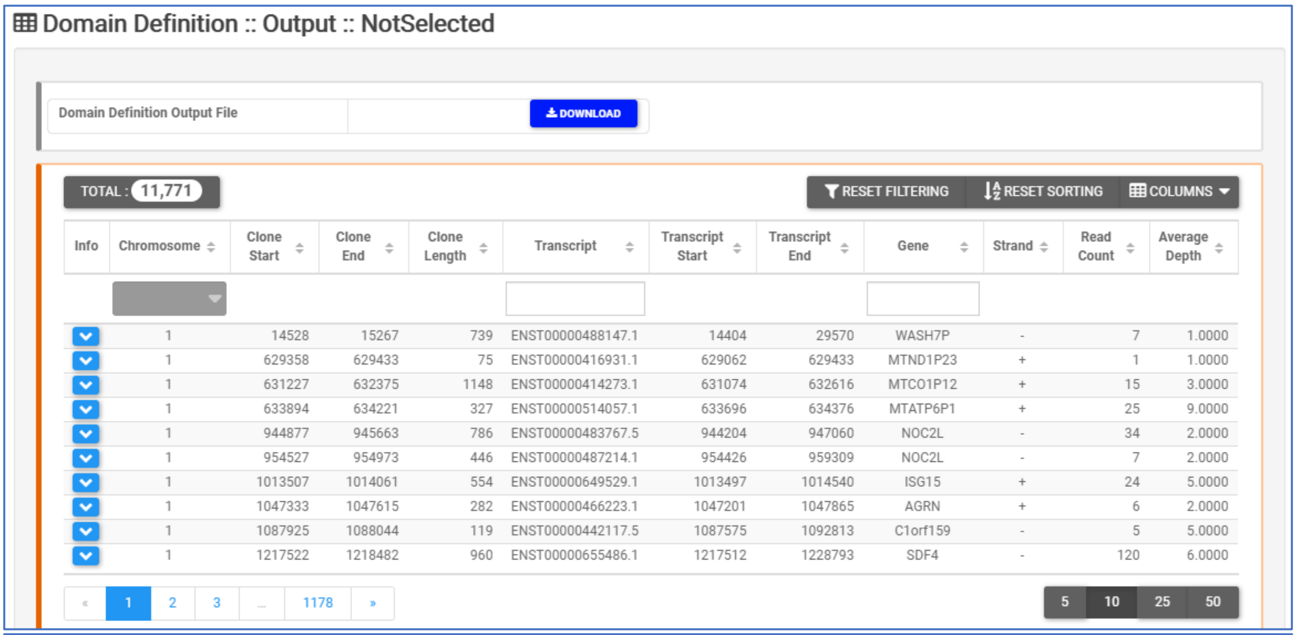

Info	Chromosome	Clone Start	Clone End	Clone Length	Transcript	Transcript Start	Transcript End	Gene	Strand	Read Count	Average Depth
	1	14528	15267	739	ENST00000488147.1	14404	29570	WASH7P	-	7	1.0000
	1	629358	629433	75	ENST00000416931.1	629062	629433	MTND1P23	+	1	1.0000
	1	631227	632375	1148	ENST00000414273.1	631074	632616	MTCO1P12	+	15	3.0000
	1	633894	634221	327	ENST00000514057.1	633696	634376	MTATP6P1	+	25	9.0000
	1	944877	945663	786	ENST00000483767.5	944204	947060	NOC2L	-	34	2.0000
	1	954527	954973	446	ENST00000487214.1	954426	959309	NOC2L	-	7	2.0000
	1	1013507	1014061	554	ENST00000649529.1	1013497	1014540	ISG15	+	24	5.0000
	1	1047333	1047615	282	ENST00000466223.1	1047201	1047865	AGRN	+	6	2.0000
	1	1087925	1088044	119	ENST00000442117.5	1087575	1092813	C1orf159	-	5	5.0000
	1	1217522	1218482	960	ENST00000655486.1	1217512	1228793	SDF4	-	120	6.0000

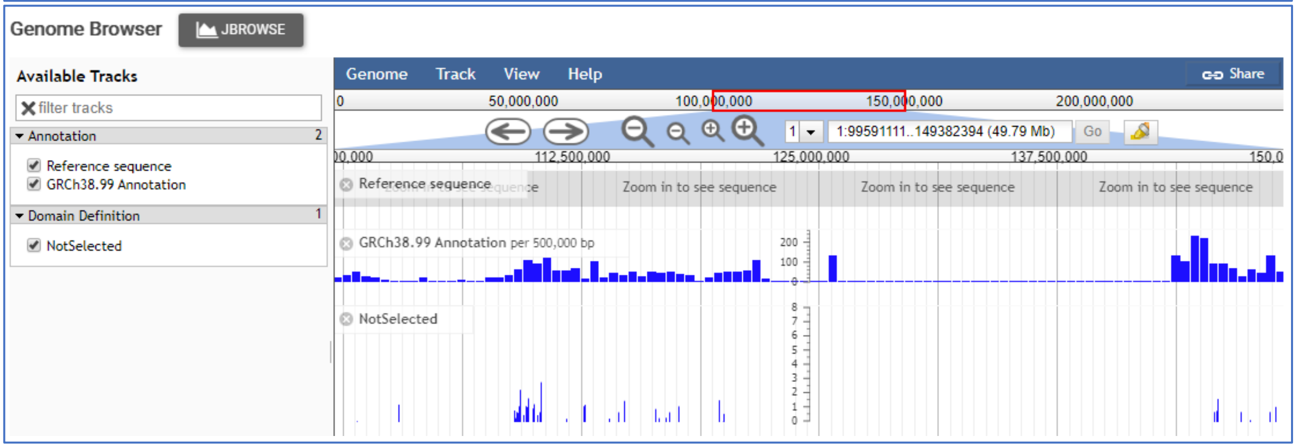

Domain Enrichment takes as input the Control and Selection output of Domain Definition step. Enrich domains for each defined domain by selecting the Genomic and the target domains. In the current example, select:

- Delta5Uchl1ssDNA and Delta5Uchl1tRNANotSelected
- invSINEB2ssDNA and invSINEB2tRNA

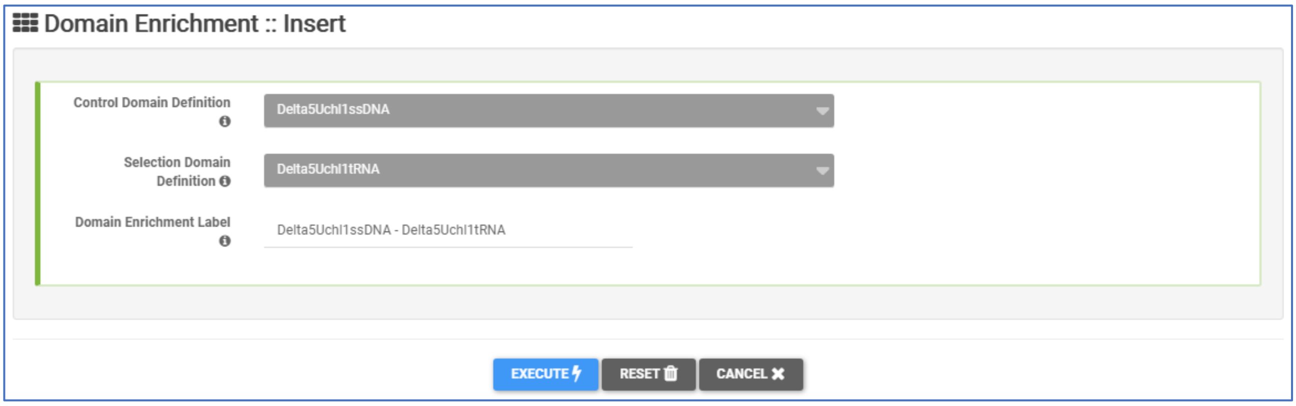

Domain Enrichment List

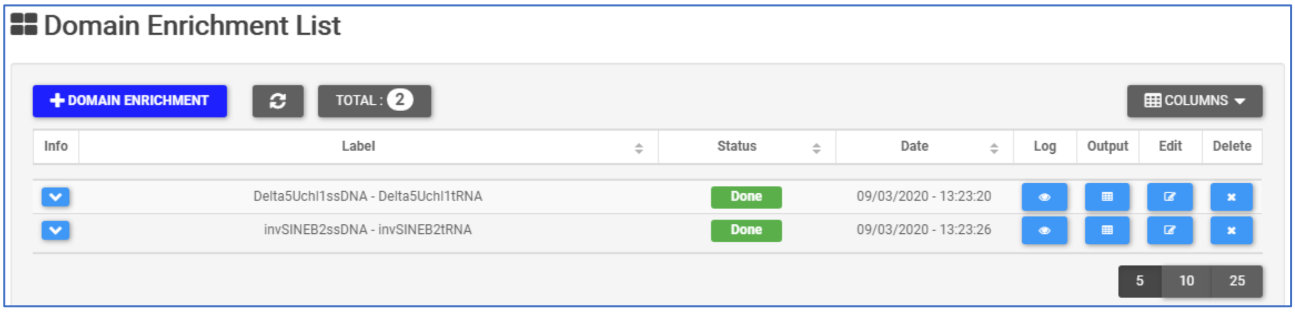

Info – Drop-down menu with information of Domain Definition input file**.**

Label – Sample label.

Status – When the execution ends successfully, the button turns green, otherwise, it turns red.

Date – Day and time of analysis execution

Log – Button that hides/opens a box with execution log file. When the execution is running, the log shows the possibility to stop it:

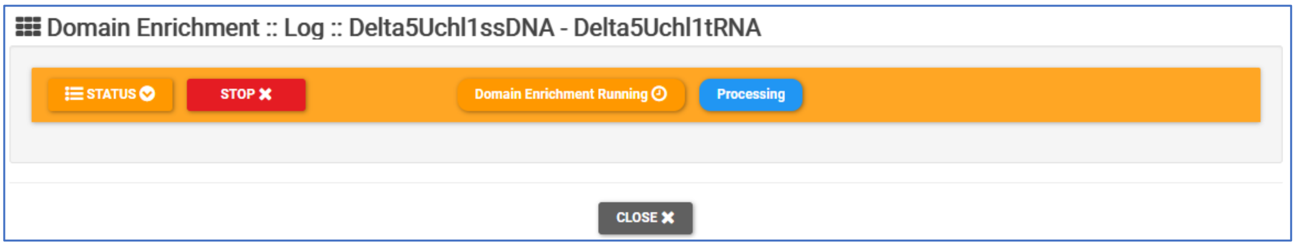

When the process has finished, the log shows summary information:

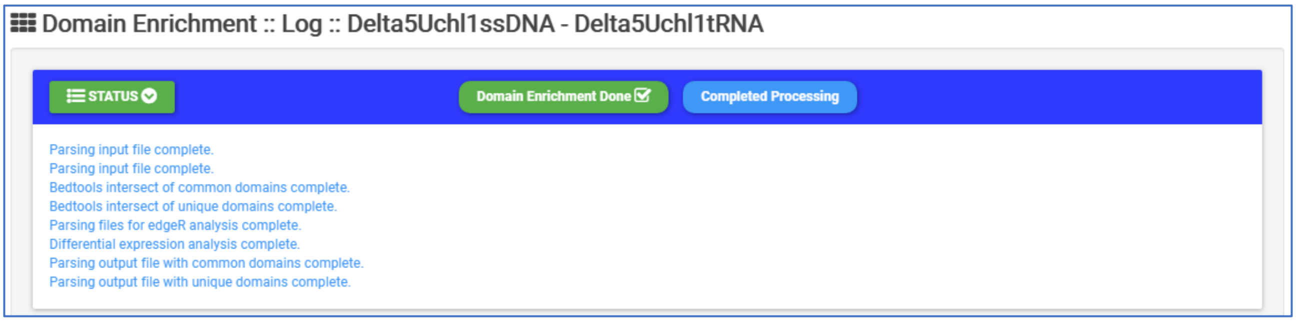

Output – Hides/opens panel with output preview

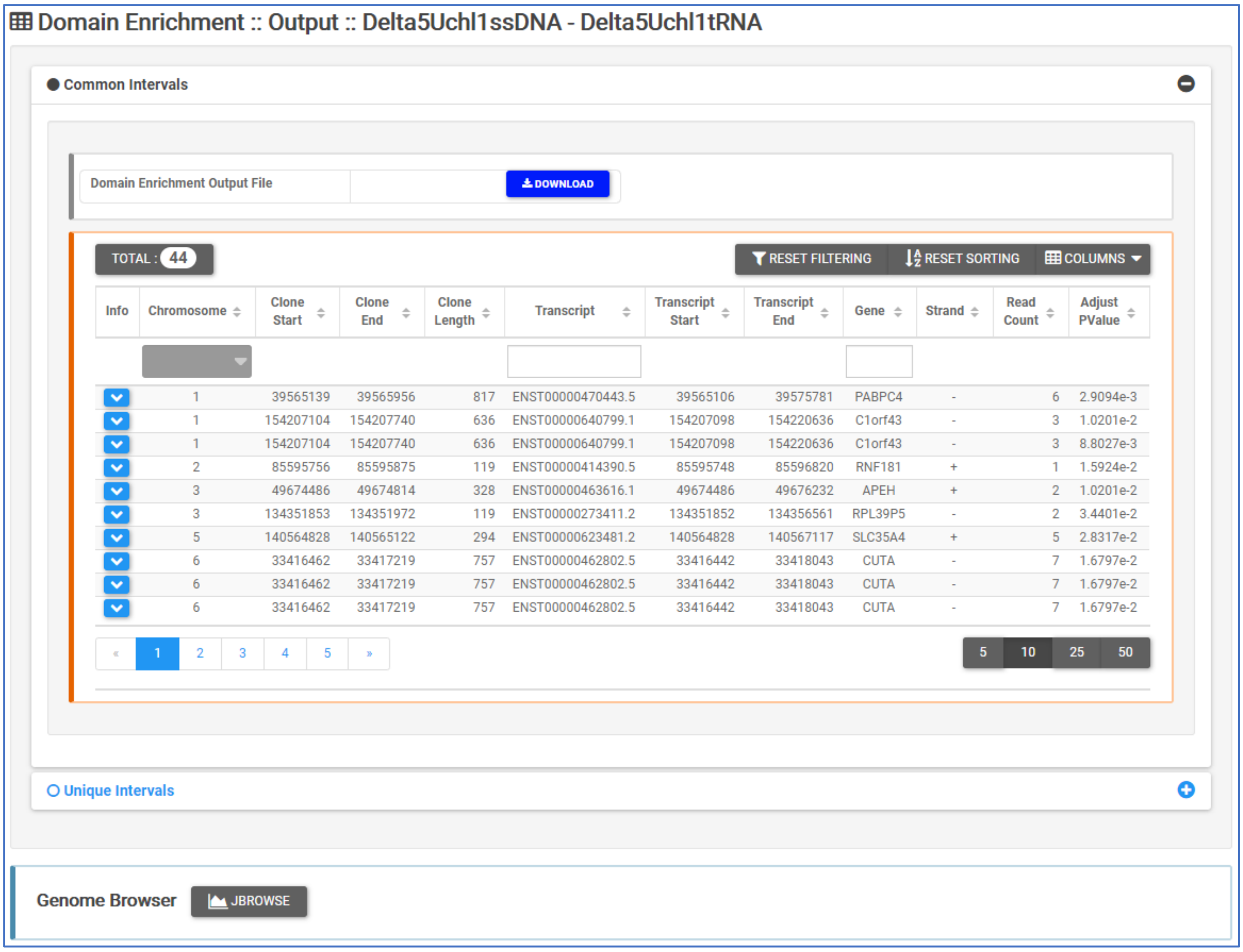

Domain Enrichment :: Output :: Delta5Uchl1ssDNA - Delta5Uchl1tRNA

Common Intervals

Domain Enrichment Output File DOWNLOAD

TOTAL : 44

RESET FILTERING RESET SORTING COLUMNS

Info	Chromosome	Clone Start	Clone End	Clone Length	Transcript	Transcript Start	Transcript End	Gene	Strand	Read Count	Adjust PValue
	1	39565139	39565956	817	ENST00000470443.5	39565106	39575781	PABPC4	-	6	2.9094e-3
	1	154207104	154207740	636	ENST00000640799.1	154207098	154220636	C1orf43	-	3	1.0201e-2
	1	154207104	154207740	636	ENST00000640799.1	154207098	154220636	C1orf43	-	3	8.8027e-3
	2	85595756	85595875	119	ENST00000414390.5	85595748	85596820	RNF181	+	1	1.5924e-2
	3	49674486	49674814	328	ENST00000463616.1	49674486	49676232	APEH	+	2	1.0201e-2
	3	134351853	134351972	119	ENST00000273411.2	134351852	134356561	RPL39P5	-	2	3.4401e-2
	5	140564828	140565122	294	ENST00000623481.2	140564828	140567117	SLC35A4	+	5	2.8317e-2
	6	33416462	33417219	757	ENST00000462802.5	33416442	33418043	CUTA	-	7	1.6797e-2
	6	33416462	33417219	757	ENST00000462802.5	33416442	33418043	CUTA	-	7	1.6797e-2
	6	33416462	33417219	757	ENST00000462802.5	33416442	33418043	CUTA	-	7	1.6797e-2

« 1 2 3 4 5 »   5 10 25 50

Unique Intervals

Genome Browser JBROWSE

Results

The summary of all the analysis performed is showed in the results page

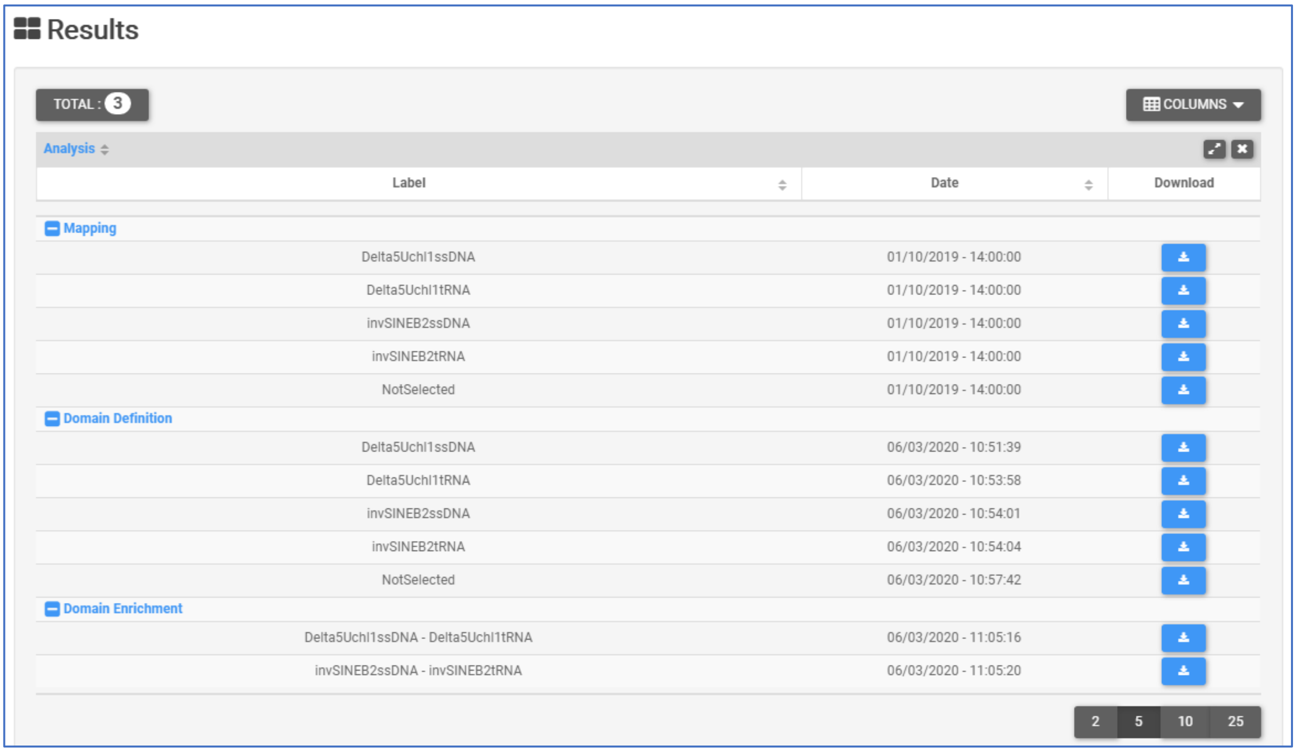

And a genome browser with the results aligned to the reference genome

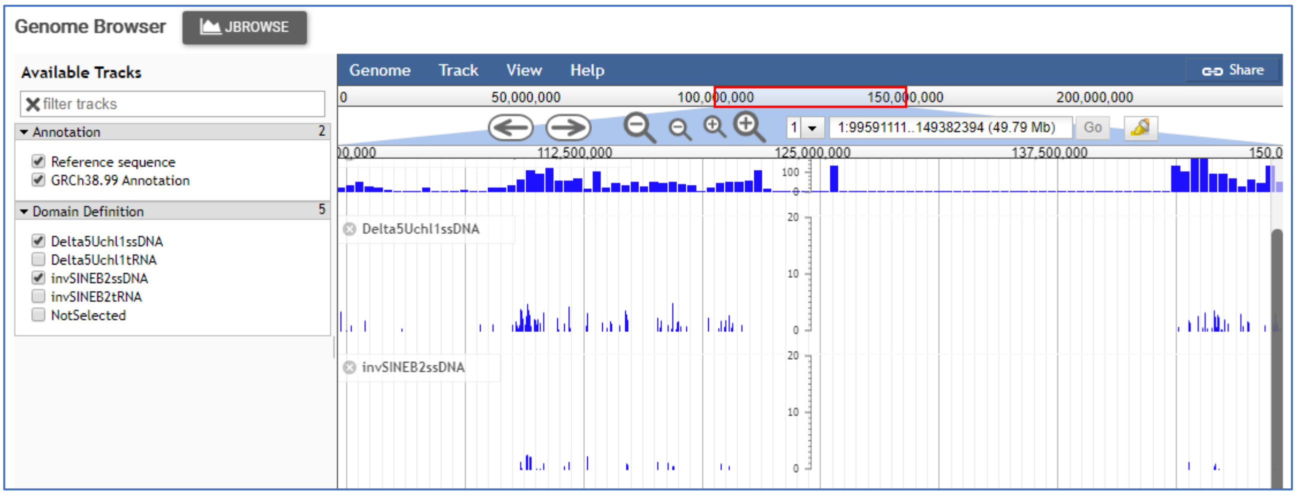

Supplement: gkaa363_Supplemental_Files [file gkaa363_supplemental_files.zip › Tutorials.pdf]
